# Probing the Role of Accessory Domains in Oxygen Stability of [FeFe]-Hydrogenases

**DOI:** 10.1021/jacs.6c05865

**Published:** 2026-07-10

**Authors:** Abdulrahman Alogaidi, Stephen B. Carr, Lucy Hudson, Henry Lloyd-Laney, Alison Parkin, Ashley Love, Michael W. George, Anca Pordea, Simone Morra

**Affiliations:** † Faculty of Engineering, 6123 University of Nottingham, University Park, Nottingham NG7 2RD, United Kingdom; ‡ Department of Chemistry, 6396 University of Oxford, Oxford OX1 3QR, United Kingdom; § Research Complex at Harwell, Rutherford Appleton Laboratory, Didcot, Oxfordshire OX11 0FA, United Kingdom; ∥ Department of Chemistry, 8748 University of York, Heslington, York YO10 5DD, United Kingdom; ⊥ School of Chemistry, University of Nottingham, University Park, Nottingham NG7 2RD, United Kingdom

## Abstract

[FeFe]-hydrogenases are the fastest enzymes for hydrogen evolution, yet their irreversible inhibition by oxygen has thwarted their industrial use. *Cb*A5H is an exception as its inhibition by oxygen is reversible. Protein scaffold rearrangement near the active site, allowing a ligand to coordinate the metal center in addition to the formation of a highly oxidized state of the metal center named H_inact_, is the current hypothesis for *Cb*A5H oxygen stability. However, the ligand identity has been disputed and there is no evidence to suggest that protein scaffold rearrangement is the sole reason for oxygen stability. Here, we investigate *Cb*A5H oxygen stability by providing a high-resolution (1.96 Å) X-ray structure that shows that the protective ligand is a conserved cysteine thiol group, which directly coordinates the metal center. The local rearrangement also encompasses structural water molecules and the side chain of E341, associated with proton transfer. In addition, we illustrate that C236 and H245, located close to accessory iron sulfur clusters in the Fd domain, influence oxygen stability. We show that mutating these residues significantly decreases oxygen stability but not the ability to form H_i__nact_. Variant C236A displays a slower inactivation rate, which we suggest is due to tuning the redox properties of one of the accessory iron sulfur clusters. We also show that the soluble ligand-binding β-grasp domain (SLBB) may not be required for oxygen stability by comparing *Cb*A5H to a novel homolog lacking this domain. Collectively, these findings expand our understanding of oxygen stability in [FeFe]-hydrogenases.

## Graphical abstract

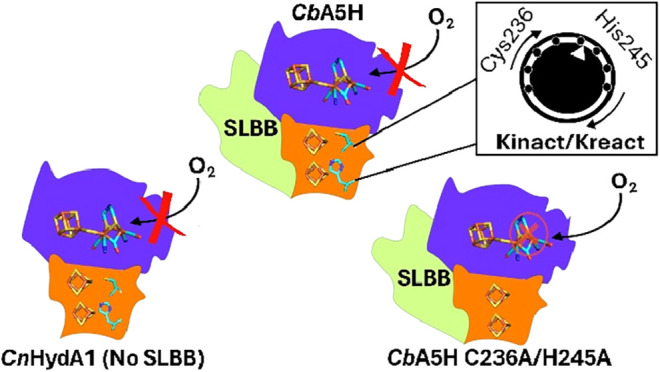

## Introduction

Hydrogenases are metalloenzymes that catalyze the reversible interconversion of H_2_/H^+^ using Earth-abundant metals, making them an attractive target for industrial applications in hydrogen production and the development of clean energy technologies. [FeFe]-hydrogenases are of particular interest due to their high rates of hydrogen production,^1–4^ but their acute oxygen sensitivity has, so far, limited their use.^5^ However, naturally occurring oxygen-protected [FeFe]-hydrogenases exist^6^ and are highly promising biocatalysts as demonstrated by *Cb*A5H from *Clostridium beijerinckii*.^7,8^

[FeFe]-hydrogenases contain a unique metal center called the H-cluster, comprised of a [4Fe4S]_H_ and [2Fe]_H_ subclusters. The two subclusters are connected by the sulfur atom of a cysteine residue (Figure 1A). The iron ions of the [2Fe]_H_ subcluster are identified as the proximal iron (Fe_p_) and distal iron (Fe_d_) based on their distance to the [4Fe4S]_H_ cluster. The two iron ions are coordinated by an azadithiolate ligand (NH­(CH_2_S)_2_^2–^, ADT for short), two cyanide ligands, and three carbon monoxide ligands.^9,10^ Fe_p_ is coordinatively saturated, while Fe_d_ has an open coordination site in the active enzyme. The open coordination position is the binding site for substrates, including H_2_ and H^+^, and inhibitors such as CO and O_2_.^11^

**Figure 1:**
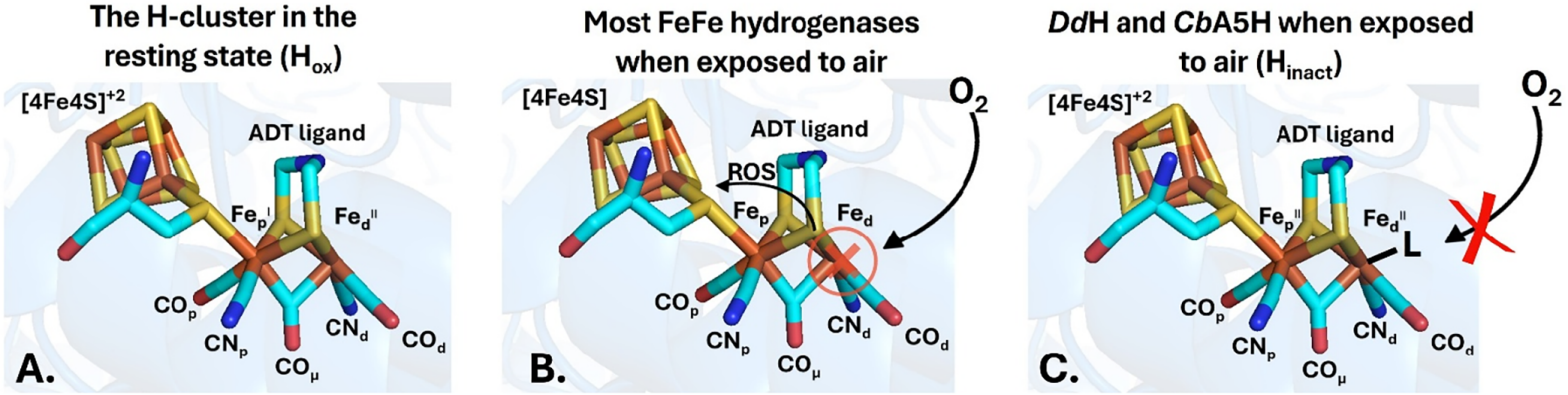
H-cluster of [FeFe]-hydrogenase at the resting redox state (H_ox_) (PDB: 6NAC) and its oxygen damage and oxygen protection mechanisms. (A) The H-cluster structure; (B) the proposed mechanism of oxygen attack in oxygen-sensitive [FeFe]-hydrogenases, (C) the oxidation of the H-cluster forming H_inact_ state in addition to the binding of a ligand (L) to the Fe_d_, which is thought to be either inorganic sulfide (in *Dd*H) or a cysteine thiolate (from C367) or a hydroxide group (in *Cb*A5H). ADT is the bridging azadithiolate NH­(CH_2_S)_2_^2–^ ligand. Subscripts “p” refer to the Fe closest to the [4Fe4S]_H_ cluster, and the ligands bound to it; subscripts “d” refer to the Fe furthest from the [4Fe4S]_H_ cluster, and the ligands bound to it; subscript μ refers to the bridging CO ligand between the two Fe atoms.

Most [FeFe]-hydrogenases suffer irreversible inactivation when exposed to oxygen.^3,12,13^ The proposed mechanism of inactivation was suggested to be the binding of oxygen to the Fe_d_, leading to a loss of the iron ion and the generation of reactive oxygen species (ROS), which may further degrade the [4Fe4S]_H_ cubane (Figure 1B).^5,14,15^

The first oxygen-stable [FeFe]-hydrogenase reported was *Dd*H, isolated from *Desulfovibrio desulfuricans*. Its oxygen stability is mediated by forming a catalytically inactive state, but it loses its stability once reductively activated.^16^ The inactive oxygen stable state was later characterized as the [2Fe]_H_ subcluster, achieving a highly oxidized redox state known as H_inact_ ^17^ (Figure 1C), a result of exogenous inorganic sulfur binding to the Fe_d_ of the metal center under oxidizing conditions.^18,19^ The [FeFe]-hydrogenase *Cb*A5H, however, displays an intrinsic oxygen protection mechanism.^20^ Here, protection is thought to be achieved through the binding of cysteine C367 thiolate to the Fe_d_ (Figure 1C), blocking O_2_ binding and allowing the enzyme to form H_inact_ without the need for exogenous sulfur.^21^

However, it has yet to be shown that formation of H_inact_ always equates to protection from oxygen. H_inact_ formation was recently reported in group B [FeFe]-hydrogenases,^22,23^ a divergent yet widespread group of enzymes. It was first characterized in *Cp*III from *Clostridium pasteurianum*,^22^ but its formation was not accompanied by oxygen stability.^24^

More recently, H_inact_ formation was reported in *To*HydA from *Thermosediminibacter oceani* ^23^ with accompanying oxygen stability. Both *Cp*III and *To*HydA1 feature an extra cysteine residue near the active site, which was suggested to bind the metal center and cause the formation of H_inact_.^23,24^ These findings suggest that H_inact_ formation is more common in [FeFe] hydrogenases than previously thought.

The crystal structure of air-exposed *Cb*A5H, refined at 2.9 Å resolution, shows that a peptide loop which carries T365, S366, and C367 (the TSC loop), close to the [2Fe]_H_ subcluster, is shifted relative to its position in *Cp*I and *Dd*H.^21^ This shift was proposed to position C367 thiolate close enough to bind the Fe_d_ and thus protect it from oxygen (Figure 1C). Residues located outside the TSC loop (L364, P386, A561) were suggested to facilitate the movement of the TSC loop by conferring flexibility and removing steric hindrance. However, the crystal structure of the enzyme in the H_inact_ state (PDB: 6TTL) positions the sulfur atom of C367 3.1 Å from Fe_d_. During the preparation of this paper, a further refinement of these crystallographic data was reported (PDB:9F47), showing a Fe_d_-S distance of 2.8 Å; however, one monomer in the structure still showed a Fe_d_-S distance of 3.2 Å.^25^ This distance is much longer than the typical sulfur-iron coordination, which is around 2.3 Å.^26,27^ This led others to hypothesize that C367 does not directly coordinate Fe_d_, and the protective ligand could be OH^–^, generated from the deprotonation of a nearby water molecule.^28,29^

Other regions of *Cb*A5H, located farther away from the H-cluster, have also been proposed to play a role in oxygen stability. For example, molecular dynamics simulations suggest that M382 may play a role in TSC loop flexibility, and mutagenesis of this residue to glutamate results in increased oxygen stability.^30^

In addition to the core domain hosting the H-cluster (named H domain), *Cb*A5H displays additional domains at its N-terminus: a ferredoxin-like (Fd) domain hosting two [4Fe4S] clusters, which is commonly found in other [FeFe]-hydrogenases, and a soluble-ligand-binding β-grasp (SLBB) domain, which is shared with only a few, thus far, uncharacterized [FeFe]-hydrogenases.^31,32^ The first published X-ray structure of *Cb*A5H (PDB: 6TTL) was only partial due to low resolution of 2.9 Å, and excluded the Fd domain and large parts of the SLBB domain. The re-refined structure (PDB: 9F47) provided an enhancement and contained the previously missing domains in the inactive form of the enzyme, alongside a 2.2 Å resolution cryo-EM structure (PDB: 8ZQD) of the enzyme in its active state (most likely H_ox_).^25^ Additionally, it was shown through X-ray fluorescence, ICP-OES, and single wavelength anomalous diffraction that the SLBB domain in *Cb*A5H contains a zinc ion, which plays a role in the dimerization of the protein.^25^

*Cb*A5H has many unique structural features that differ from typical oxygen-sensitive [FeFe]-hydrogenases. Whether these structural features are playing additional role(s) in oxygen stability is unclear, precluding rational approaches for stability improvement. In this study, we further dissect the *Cb*A5H mechanism(s) of oxygen stability by combining a higher resolution X-ray crystal structure of *Cb*A5H in the H_inact_ redox state, site-directed mutagenesis, and comparison to a new *Cb*A5H-like enzyme (*Cn*HydA1). We show that the C367 and Fe_d_ are separated by 2.5 Å, more consistent with direct Fe–S coordination. We also show that the protein environment of the metal clusters in the Fd domain is crucial for oxygen stability, while the SLBB domain may not be required for oxygen stability.

## Results

### Characterizing the Oxygen-Protected *Cb*A5H: A High-Resolution Structure

In order to investigate the structural features of *Cb*A5H, we matured the enzyme *in vivo* with HydEFG maturases as previously reported,^8^ and removed the affinity tag after aerobic purification. Following size exclusion chromatography, the enzyme was crystallized anaerobically, resulting in crystals that diffracted to a resolution of 1.96 Å. This allowed accurate modeling of the full polypeptide chain, metal centers, and water molecules.

The crystals contain a dimer in the asymmetric unit with the H- and SLBB-domains forming the dimeric interface (Figures 2 and S6 for elaboration), as previously shown.^21,25^ It was possible to model all residues in the enzyme except 10 (chain A is missing G2-K5, L28-N30, and K642-D644, while chain B is missing G2-K5, N30, N100, I101, and K642-D644), providing a significant improvement over the previous crystal structure (PDB: 6TTL). The final refined model displays excellent geometry and has an R factor of 0.184 and R free of 0.204 (Table S4). The significant increase in resolution allowed more accurate modeling of the active site environment, revealing the distance between C367 and the distal iron to be 2.5 Å (Figure 2A), which supports direct coordination of a sulfur atom as the mechanism of oxygen stability.

**Figure 2:**
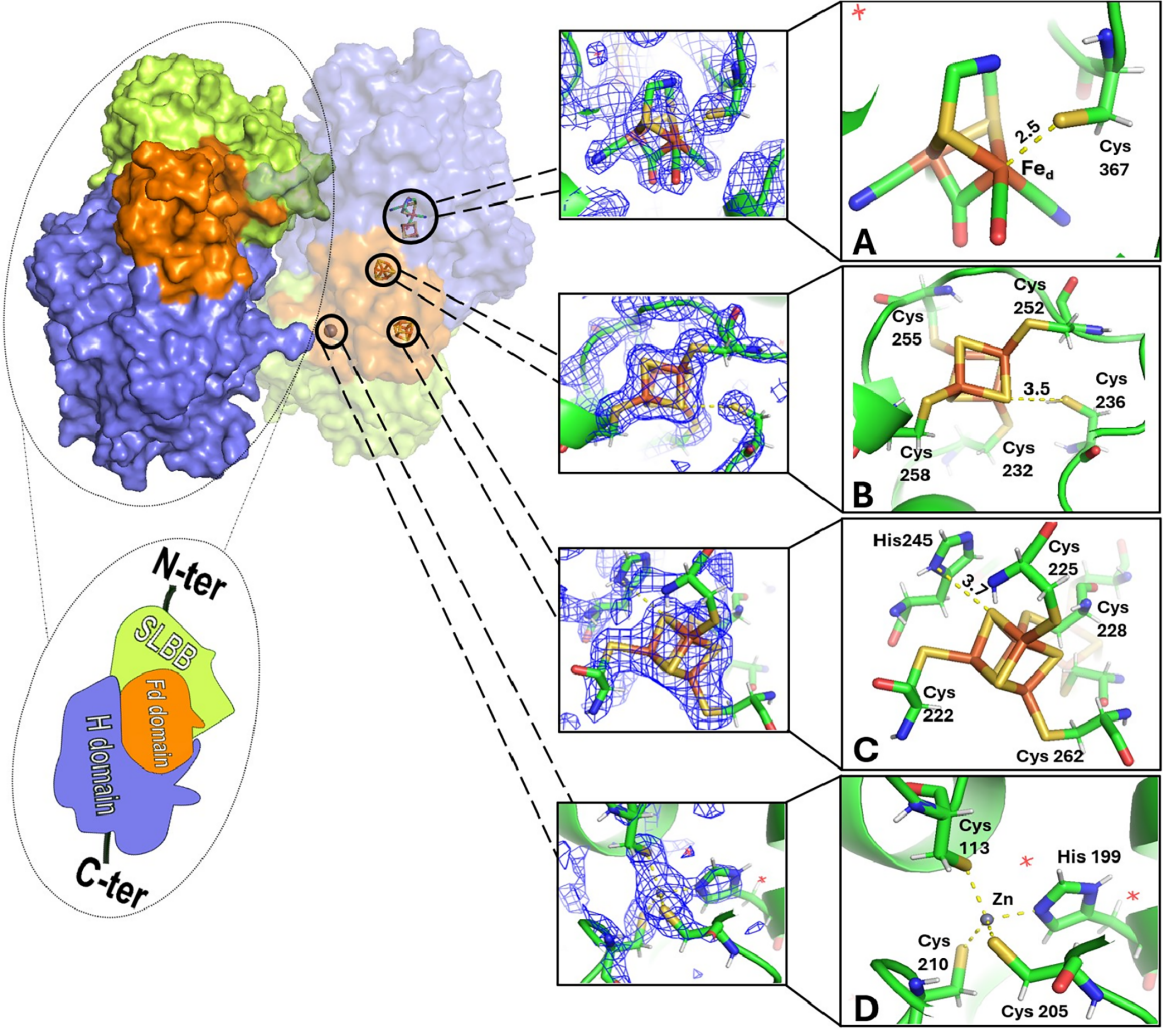
X-ray structure of *Cb*A5H in the H_inact_ state (PDB:9RKM). The main figure shows the overall structure of the protein dimer, with one monomer being transparent to distinguish between the two monomers and to allow the visualization of the metal cofactors. Each domain is color-coded: H domain (blue), Fd domain (orange), SLBB domain (green). (A) The H-cluster environment reveals C367 within direct coordination distance of Fe_d_. (B) The proximal iron sulfur cluster in the Fd domain shows the location of C236 and its distance to the cluster. (C) The distal iron sulfur cluster in the Fd domain shows the location of H245 and its distance to the cluster. (D) The zinc ion and its coordinating residues in the SLBB domain. Electron density maps adjusted to 2σ.

The improved resolution also allowed the modeling of ordered water molecules, including two structural waters near the H-cluster that are likely part of the proton transfer pathway (PTP) (Figure S7). The PTP is essential for shuttling protons to and from the H-cluster and is composed of ordered water molecules and conserved residues.^33–35^ The side chain of E341 in *Cb*A5H and the two water molecules are significantly shifted in position when compared with both *Cp*I and *Dd*H (Figure S7A,B). Comparing the active site architecture of *Cb*A5H with *Cp*I led Winkler and co-workers to hypothesize a network of hydrogen bonds that stabilize the TSC loop in the H_inact_ conformation.^21^ Our structure provides exact details of this H-bonding network (Figure S7C), which includes residues I564, S366, W371, E341, K390, and Y637 and five structural water molecules.

The crystal structure confirmed that the Fd domain has two [4Fe4S] clusters, as predicted by the protein amino acid sequence: one close to the H domain (the proximal cluster), and the other further (distal cluster). Both showed cubane-type geometry and are coordinated by four cysteine residues (C232, C252, C255, and C258 for the proximal, and *C*222, C225, C228, and C262 for the distal cluster, see Figure 2). The protein dimer is arranged to ensure the exposure of the Fd domain to the surface. This aligns with the notion that the Fd domain’s primary role is electron transfer, where surface exposure would be necessary to interact with cellular redox partners.

To confirm the identity, structure, and position of metal cofactors in the protein, anomalous diffraction data were collected using X-rays with wavelengths close to the k-edges of zinc and iron. The results supported that each iron–sulfur cluster in the Fd domain contained four Fe ions (Figure S8). The data also confirmed the presence of one zinc ion coordinated by H199, C205, C113, and C210 in the SLBB domain (Figures 2 and S9 for anomalous difference map), supporting the findings of Duan et al.^25^ A further 14 iron ions are present in each monomer of the protein: four in each of the distal and proximal iron–sulfur clusters in the Fd domain and six in the H-cluster (Figure S8). In addition, the refined occupancy of the metal cofactors, including the H clusters, was 100%. This is particularly interesting as the enzyme was matured *in vivo*, using *Clostridium acetobutylicum* maturases HydEFG, coexpressed with the enzyme. This demonstrates that the maturases are highly efficient in assembling and inserting the H-cluster, even though the target enzyme is from a different species and all proteins are overexpressed heterologously in *Escherichia coli*.

### Ferredoxin Domain is Key for Oxygen Stability in [FeFe]-Hydrogenases

It was previously suggested that the Fd domains may increase oxygen stability of [FeFe]-hydrogenases by shuttling electrons to the active site to counteract oxidative damage;^36,37^ however, this was dismissed when Caserta et al. showed that the removal of this domain from the relatively oxygen-stable *Me*HydA ([FeFe]-hydrogenase from *Megasphaera elsdenii*) did not affect its oxygen stability.^38^

Our X-ray structure shows polar residues close to the iron–sulfur clusters of the Fd domain. For example, the proximal cluster features a fifth cysteine (C236), positioned 3.5 Å from a sulfur atom in the cluster but does not appear to be in direct coordination (Figure 2B). Likewise, the distal cluster features a noncoordinating histidine (H245), which is again near one sulfur atom (3.7 Å) of the cluster (Figure 2C). It is well established that second sphere polar residues that are not directly coordinating an iron sulfur cluster can tune its electronic properties. Site-directed mutagenesis has shown that changing the polarity of such residues can significantly alter a cluster’s potential.^39–42^

We hypothesize that the electronic properties of the Fd domain clusters may play a role in the oxygen stability of *Cb*A5H. Given that the two polar residues (C236 and H245) are at a distance that may allow them to modulate the redox potential of the clusters, we hypothesize that they could be linked to the enzyme’s oxygen stability. Additionally, multiple sequence alignment (Figure S11) revealed that C236 and H245 are not conserved in oxygen-sensitive [FeFe]-hydrogenases, such as *Cp*I, while H245 is conserved in the oxygen-stable *Dd*H (H58). This observation suggests that H245, and to a lesser extent C236, may be important for oxygen stability. Structural alignment of the Fd domains of *Cb*A5H and the oxygen-sensitive *Cp*I (*Cp*I PDB: 6N59) showed that C236 and H245 in *Cb*A5H correspond to A165 and I177 (hydrophobic residues) in *Cp*I, respectively (Figure S12). Consequently, we chose to mutate C236 and H245 to alanine.

We expressed and purified three *Cb*A5H variants, C236A, H245A, and C236A/H245A, in addition to the oxygen-sensitive *Ca*HydA1 [FeFe]-hydrogenase from *C. acetobutylicum* for comparison. Each was tested for its specific activity in hydrogen evolution under three conditions: (1) when the enzyme was handled strictly anaerobically, or after exposing the samples to air for (2) 4 h, or (3) 24 h (Figure 3). The activity of C236A, H245A, and C236A/H245A variants after 4 h of air exposure was significantly lower than that of the wild-type enzyme. H245A and C236A/H245A showed oxygen stability levels comparable to that of the oxygen-sensitive *Ca*HydA1 measured under the same conditions. The same pattern of activity loss was also observed after 24 h of air exposure.

**Figure 3:**
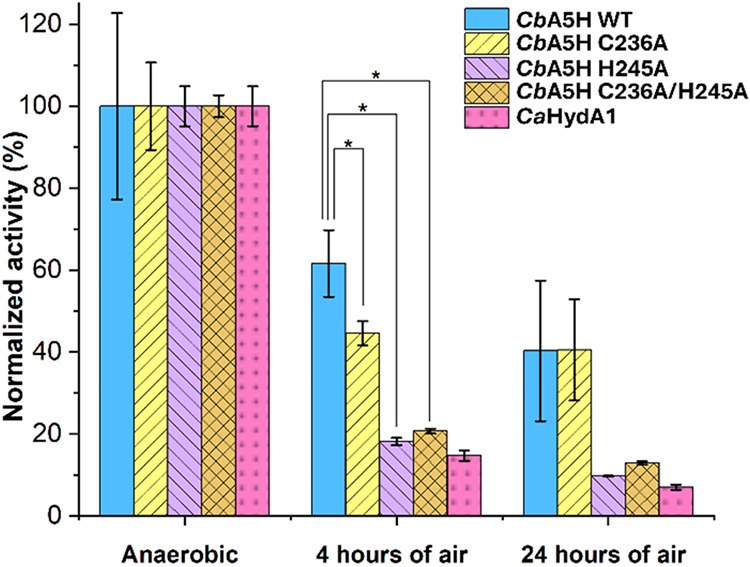
Retained H_2_ evolution activity of *Cb*A5H WT, *Cb*A5H C236A, *Cb*A5H H245A, *Cb*A5H C236A/H245A variants, and CaHydA1 after either 4 or 24 h of air exposure. Data is normalized to the average activity measured for enzyme samples, which were not exposed to oxygen (anaerobic). See Table S5 for specific activity before normalization to 100%. Asterisk represents a p-value ≤ 0.05.

We also characterized the redox state of the H-cluster in *Cb*A5H WT and the three variants using Fourier transform infrared (FTIR) spectroscopy at three time points: (1) when the enzyme is handled anaerobically, (2) after 30 min of air exposure, and (3) after 4 h of air exposure (Figure 4). The anaerobic FTIR spectrum of *Cb*A5H WT shows a mixture of redox states dominated by H_ox_ and H_red_H^+^, as reported previously.^20,21^ The anaerobic FTIR spectra of the three variants were very similar to that of the WT, suggesting that the mutations are not affecting the structure of the active site and ruling this out as a reason for the observed activity loss. After 4 h of air exposure, all variants displayed the characteristic H_inact_ spectral fingerprint of the WT, once again supporting that there is no major impact on the H-cluster structure. Interestingly, variants C236A and C236A/H245A did not form a pure H_inact_ enzyme sample within 30 min of air exposure, unlike *Cb*A5H WT and H245A. In other words, after 30 min of air exposure, C236A and C236A/H245A variants showed a mixture of H_inact_ and H_ox_. While this type of experiment does not allow an exact determination of inactivation kinetics, it suggests that the rate of forming H_inact_ was affected by the C236A mutation.

**Figure 4:**
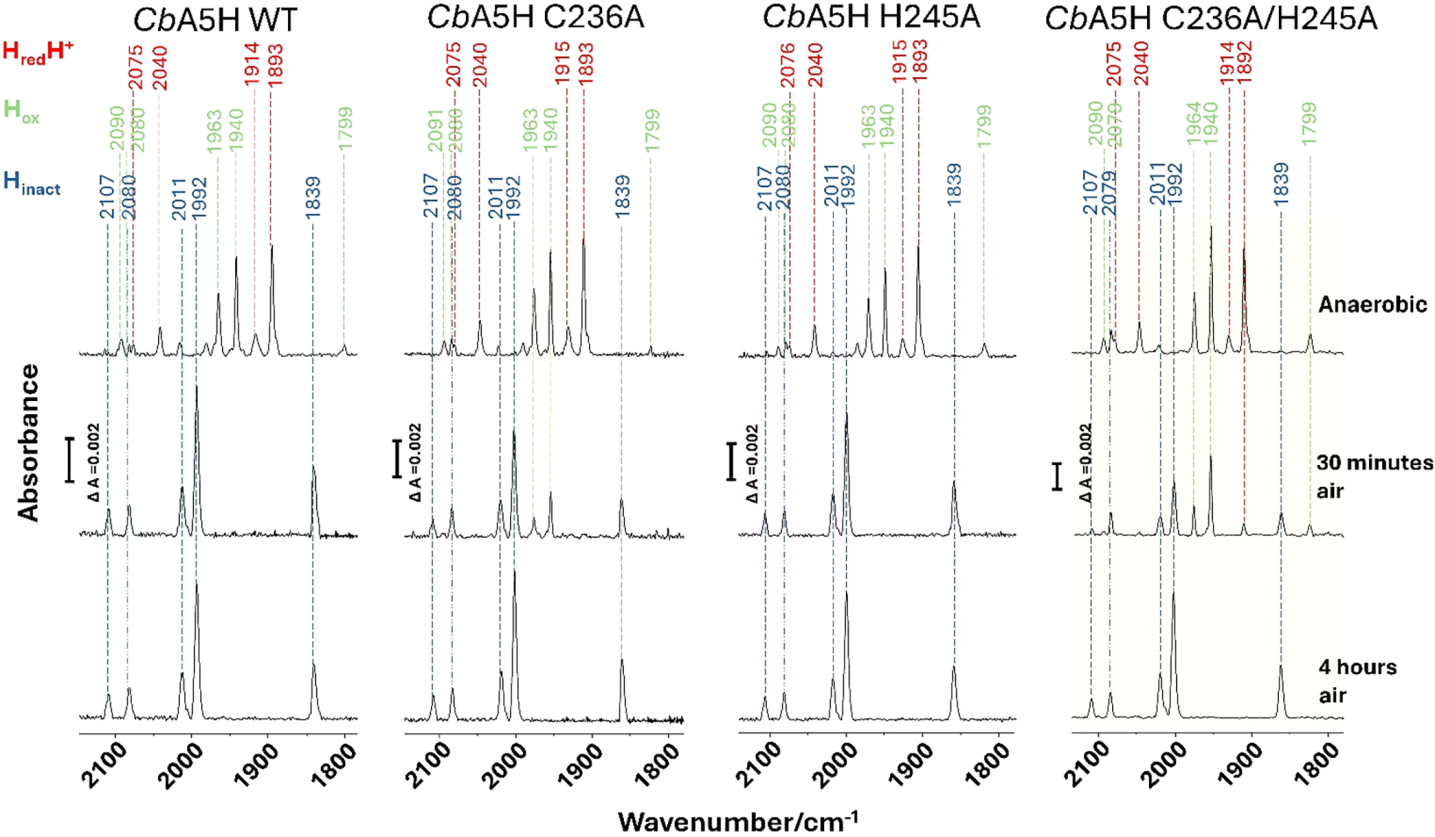
FTIR spectra of *Cb*A5H WT, *Cb*A5H C236A, *Cb*A5H H245A, and *Cb*A5H C236A/H245A measured when samples were handled anaerobically, after 30 min of air exposure, and after 4 h of air exposure. The spectral signature of the H-cluster was monitored in the range of 2150–1750 cm^–1^, where 2150 to 2025, 2025 to 1875, and 1875 to 1795 cm^–1^ are the typical absorbance regions of CN_p/d_, CO_p/d_, and CO_μ_, respectively. Peaks were assigned to redox states according to previous reports.^20,21,29^

We further characterized the *Cb*A5H WT and variants using protein film electrochemistry. Oxygen-injection experiments recapitulate the results shown in Figures 3 and 4, with the WT enzyme showing a greater recovery from aerobic exposure than all three of the variants (Figure S13). Again, between the variants, H245A shows the lowest recovery from oxygen exposure and C236A has the highest recovery.

Under oxygen-free conditions, cyclic voltammograms at pH 8.0, 100% H_2_ (Figures 5A, S14, and S15) show no discernible change in onset potential at pH 8.0, confirming that the mutations have not influenced the bidirectional catalytic activity of *Cb*A5H. This was also evident when cyclic voltammetry comparisons of the WT and variants were conducted at pH 8.0, 100% N_2_ and at pH 6.0, 100% H_2_, conditions under which H_2_ production activity is enhanced (Figures S16 and S17). Again, there is no significant change in the catalytic bias. Thus, our electrochemical results complement the anaerobic H_2_ evolution assay data (Figure 3) and the FTIR (Figure 4), confirming that the mutations did not change the H-cluster structure or catalytic reactivity.

**Figure 5:**
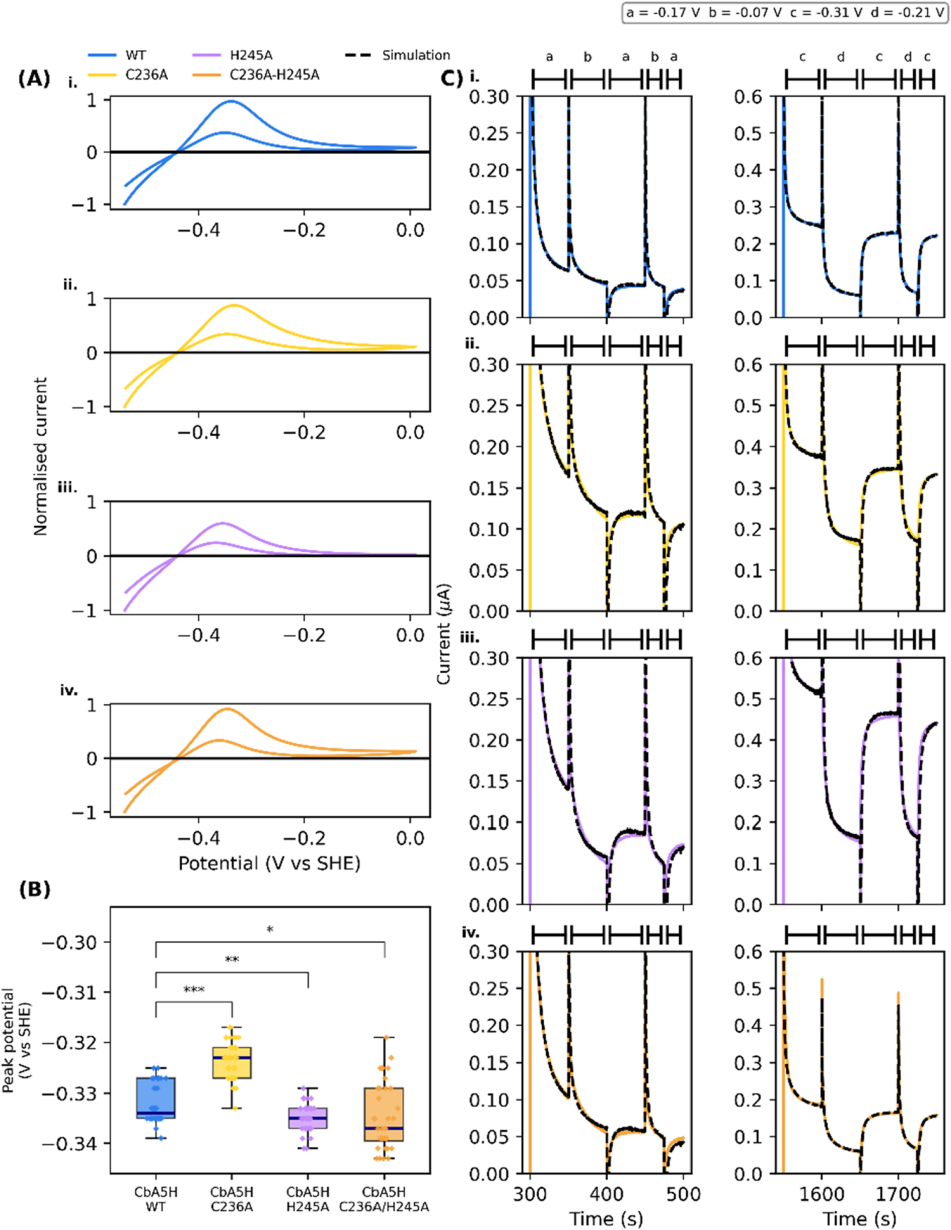
(A) Scan 1 of *Cb*A5H 3 mV s^–1^ cyclic voltammetry measurements with the current normalized to the starting value. (i) WT (blue); (ii) C236A (yellow); (iii) H245A (purple); and (iv) C236A/H245A (orange). Experimental repeats shown in Figure S15. (B) Box plots of maximal H_2_ oxidation current peak potentials for *Cb*A5H WT and variants. Source data set shown in Figure S15, 36 data points (◆) per enzyme. Asterisks denote results of two-sample *t*-test where * = 0.01 < *p* < 0.05, ** = 0.001 < *p* < 0.01, and *** = 0 < *p* < 0.001. (C) The potential-time and blank-corrected, normalized current response, and corresponding simulation, when stepping potential from −0.285 to −0.185 V vs SHE (phase 1 of the anaerobic oxidative inactivation/reductive reactivation experimental procedure, full data sets shown in Figures S19–S22). All experiments were conducted at pH 8.0, 100% H_2_, 5 °C, electrode rotation rate 3,000 rpm.

As shown in Figure 5A, the characteristic high potential, reversible oxidative inactivation, attributed to the formation of the H_inact_ state in *Cb*A5H WT, is clearly visible in electrochemical experiments on all variants. Analysis of the peak potential extracted from 3 repeats of continual voltammetric cycling experiments (Figure S15) showed a small but statistically significant difference in the value extracted for all three variants relative to the WT enzyme. We confirmed that this could not be attributable to changes in sensitivity to Cl^–^ inhibition via continuous scanning experiments in the absence and then presence of NaCl (Figure S18).

In these chloride-addition experiments, there is no change in the extent of high-potential inactivation or the potential window of inactivation, and the chloride-free data confirm that the electrochemically induced oxidative inactivation can therefore be solely attributed to the formation of the H_inact_ state.

The positive shift in the peak potential of C236A could indicate slower formation of H_inact_, as was observed with FTIR; in order to quantify this more accurately, potential step chronoamperometry was performed using the methodology first described by Winkler and co-workers^21^ (Figure S19). In these experiments, the enzyme is inactivated by stepping the working electrode to an oxidizing potential, without the addition of oxygen or air. The current–time response was analyzed using the three-state A_1_ ⇄ A_2_ ⇄ I model described previously.^21^ When attempting to use the mathematical model to recover information from the chronoamperometry data, there are numerous modeling choices that must be made that relate to trade-offs between accurately describing the data and recovering kinetic information in a principled manner. We have outlined these decision points in the SI. Our ultimate solution was to obtain parameter estimates for each inactivation/reactivation H_2_-oxidation “phase” of the experiment (the parts of the experiment separated by potential pulses to −0.54 V) as separate optimizations. We then used Markov chain Monte Carlo (MCMC) methods to quantify the parameter uncertainty arising from the fit between simulation and data. These results are summarized in Table 1. Although the sensitivity to the actual parameters is high on a per-phase basis (as indicated by the small value of the standard deviation), there is significant variation in the recovered parameters both between phases and between repeats. However, when comparing the WT *k*_inact_ value with that of the C236A variant, both the mean and the 20th–80th percentile region are substantially lower in the C236A case. Something similar can be said for the H245A and C236A-H245A variants, which have a substantially higher value for *k*_react_ when compared to the WT enzyme. These observations support the hypothesis that formation of H_inact_ is slower for C236A, and the rate of reactivation from H_inact_ is higher in variants with a H245A mutation. This agreement with the spectroscopy results, coupled with the independent recovery of the parameter values for *k*_inact_, *k*_–1_, and *k*_1_ WT values reported by Winkler et al.^21^ lend credence to the computational analysis of the electrochemical data. Thus, these results support the notion that the ferredoxin domain can tune the inactivation and reactivation rates of the H-cluster in *Cb*A5H.

**Table 1:**
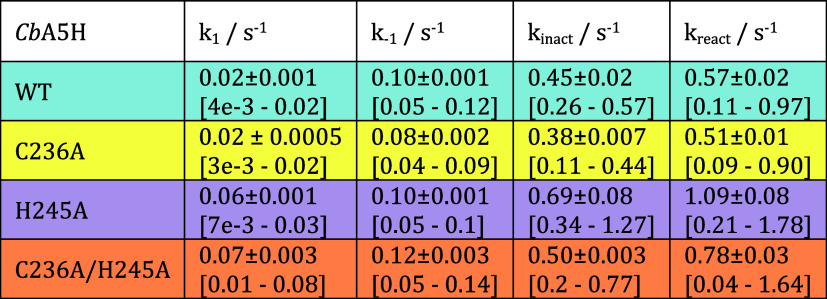
Rate Constants Determined from Markov Chain Monte Carlo (MCMC) Simulations of Electrochemical Inactivation/Reactivation Potential Step Experiments (Figures 5C and S19–S22)­^a^

a Using the inactivation model *A*_1_ ⇄ *A*_2_ ⇄ I, *k*_1_ describes the rate of *A*_1_ → *A*_2_; *k*_–1_ describes *A*_2_ → *A*_1_; kinact describes *A*_2_ → I; *k*_react_ I → *A*_2_. Values are reported as the mean of the posterior parameter distribution across each repeat and phase of the experiment ± the average standard deviation of the parameter distribution. The values of the 20th and 80th percentiles of the MCMC-recovered mean values are reported in square brackets.

### Role of the SLBB Domain in Oxygen Stability: *Cn*HydA1 as a Case Study

The SLBB domain in *Cb*A5H is a unique case among [FeFe]-hydrogenases. While truncating domains has been successful for *Me*HydA^38^ and *Ca*HydA1,^43–46^ it has been previously demonstrated that truncating the SLBB domain (or the SLBB and Fd domain simultaneously) from *Cb*A5H results in a nonfunctional enzyme.^47^ To alleviate this limitation, we attempted to generate a truncated version of *Cb*A5H fused to a GST tag, which resulted in poor expression and no activity (data not shown).

Consequently, we decided to utilize a radically different approach, i.e., identifying a naturally occurring [FeFe]-hydrogenase with similarity to *Cb*A5H but lacking the SLBB domain. *Cn*HydA1 (accession number: WP_011721785.1) is an [FeFe]-hydrogenase from *Clostridium novyi* that has a sequence identity of 46.7% to *CbA5H* and sits 7 nodes away in the phylogenetic tree (Figure S23). *Cn*HydA1 displays a different modular structure to *Cb*A5H (Figure 6A), containing only the H domain and an Fd domain with two iron–sulfur clusters, but lacking an equivalent of the SLBB domain as inferred from sequence analysis (Figure S11).

**Figure 6:**
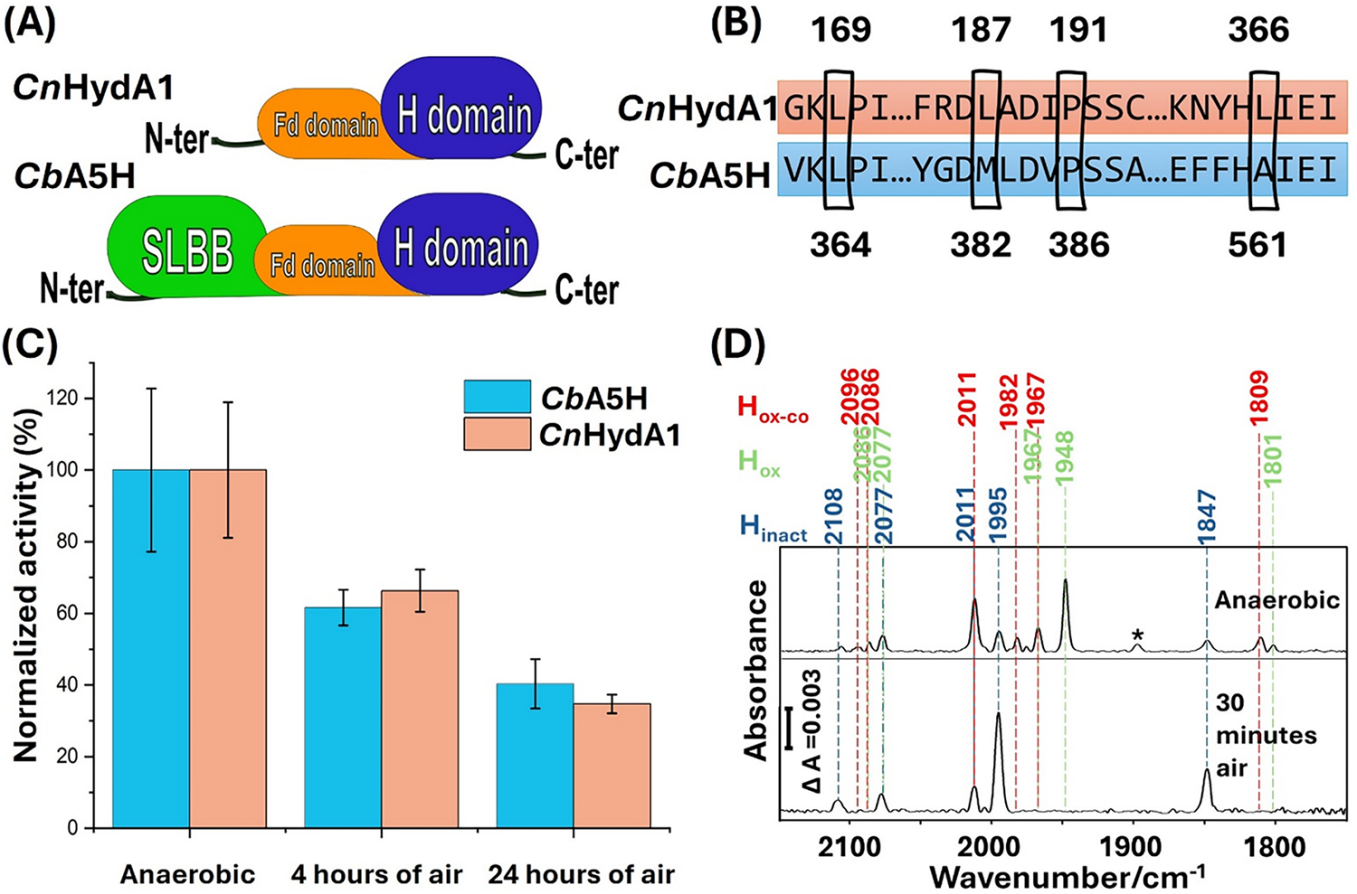
*Cn*HydA1 comparison to *Cb*A5H WT. (A) Schematic representation of the modular structure of *Cn*HydA1 and *Cb*A5H. (B) Sequence alignment of the two enzymes, highlighting residues associated with oxygen stability. (C) Specific activity of hydrogen evolution of *Cn*HydA1 and *Cb*A5H, normalized to 100%, where 100% is the average of the anaerobic activity, measured under strict anaerobic conditions, after 4 h of air exposure, and after 24 h of air exposure (see also Table S5 for specific activities). (D) the FTIR spectra of *Cn*HydA1 when purified anaerobically and after 30 min air exposure in the range of 2150–1750 cm^–1^, where 2150 to 2025, 2025 to 1875, and 1875 to 1795 cm^–1^ are the typical absorbance regions of CN_p/d_, CO_p/d_, and CO_μ_, respectively. * indicates a peak that strongly resembles H_red_H^+^.

Despite the phylogenetic proximity to *Cb*A5H, *Cn*HydA1 displays interesting sequence differences (Figure S11). With respect to the residues suggested to confer TSC loop flexibility,^21,30^ L364 and P386 in *Cb*A5H are conserved in *Cn*HydA1 (L169 and P191), while A561 and M382 in *Cb*A5H are replaced by L366 and L187, respectively (Figure 6B).

Interestingly, sequence analysis among *Cn*HydA1, *Cb*A5H, and the oxygen-sensitive *Cp*I hydrogenase shows high sequence identity among the three enzymes (Table S6). The H domain is very similar between all three enzymes, which is coherent with its primary role of hosting the H-cluster. The Fd domain in *Cn*HydA1, however, appears to be more similar to *Cb*A5H than *Cp*I (Table S6). As the Fd domain is surface-exposed in *Cb*A5H and was shown to be crucial for electron transfer, the variability may be a function of phylogenetic proximity and possibly involved in determining the affinity to species-specific redox partners. However, it may also be that the Fd domain carries features that are crucial for oxygen stability. Multiple sequence alignment also showed that C236 and H245 in *Cb*A5H are conserved in *Cn*HydA1 as C47 and H56, respectively (Figure S11), which strengthens the hypothesis that these residues are important in conferring oxygen stability.

*Cn*HydA1 was heterologously overexpressed in *E. coli* BL21­(DE3) ΔIscR^48^ alongside maturase genes hydE, hydF, and hydG from *C. acetobutylicum*.^43^ The protein was purified by affinity chromatography, yielding 8 mg protein per L of bacterial growth and with high purity (Figure S24).

To test if the enzyme was oxygen stable, it was expressed and purified anaerobically, and specific hydrogen evolution activity was tested as described for *Cb*A5H, with the enzyme either maintained under strict anaerobic conditions or exposed to air for 4 or 24 h. *Cn*HydA1 was as oxygen stable as *Cb*A5H when exposed to air for up to 24 h (Figure 6C).

The enzyme was further characterized by FTIR spectroscopy. The anaerobic FTIR spectrum revealed a mixture of redox states dominated by H_ox_, with contributions from H_red_H^+^, as well as H_ox‑CO_ and H_inact_ (Figure 6D). The spectral signatures of redox states H_ox_ and H_inact_ were assigned by comparison with spectra of *Cb*A5H (Table S7), while H_ox‑CO_ was identified after treatment with carbon monoxide (Figure S25). More importantly, the enzyme showed complete conversion to H_inact_ after 30 min of air exposure, further confirming that its stability when exposed to oxygen is not significantly affected when the SLBB domain is absent. Furthermore, *Cn*HydA1 can be conveniently purified aerobically, like *Cb*A5H, without any negative impact on activity and showing the FTIR spectral signature of H_inact_ (Figure S26). Collectively, these findings show that *Cn*HydA1 is as oxygen-stable as *Cb*A5H, suggesting that the SLBB domain may not be necessary for oxygen stability.

## Discussion

Here, we provide a high-resolution (1.96 Å) and more complete X-ray structure of *Cb*A5H in the H_inact_ state, which clearly shows the thiol of C367 to be in a plausible distance (2.5 Å) to form a direct coordination bond to the distal iron of the H-cluster. This strongly supports a mechanism of oxygen stability by direct coordination of a sulfur atom to the active site cluster,^21,25^ and contrasts the alternative hypothesis that a hydroxide might be the coordinating ligand.^29^

The local rearrangement of structural water molecules and the side chain of E341 indicate a broader structural change in the H-cluster pocket necessary to permit the binding of the thiol group of C367 to Fe_d_, while maintaining the integrity of the proton transfer pathway. The reorientation of the E341 side chain is not accompanied by a change in the protein backbone, suggesting that the different rotamer is sufficient to accommodate the change in local hydrogen bonding caused by the repositioning of the thiol group of C367 to form H_inact_. This structural change is not observed in sulfide-dependent H_inact_ of *Dd*H (Figure S7B), highlighting how structural flexibility is crucial for oxygen stability by endogenous cysteine in *Cb*A5H and may also have implications for the inactivation/reactivation process. We note that a backbone movement in the corresponding glutamate residue of oxygen-sensitive *Cr*HydA1 (E141) during O_2_ attack has been previously reported based on spectroscopic analysis of amide bands.^49^ This does not match our observation of a side chain movement in *Cb*A5H E341.

Furthermore, our crystal structure shows that the SLBB domain plays an important role in configuring the enzyme as a homodimer, with a zinc atom likely to play a structural role within the domain.^25^ We also confirm the identity of the zinc ion via anomalous diffraction, supporting the recent findings of Duan and co-workers.^25^

The structure also shows that both the proximal and distal iron sulfur clusters in *Cb*A5H are of the [4Fe4S] type. Although C236 does not directly coordinate the proximal cluster, it is 3.5 Å away from one of its sulfur atoms, a distance that may permit second sphere interaction to tune its potential. Iron sulfur clusters with supernumerary cysteines have been proposed to play a key role in [NiFe] hydrogenases’ oxygen stability, via coordinating an unusual [4Fe3S] cluster, which delivers an additional electron to the metal center and allows the complete reduction of oxygen to water.^50,51^ An unassigned EPR signal in *Cb*A5H (*g* = 2.019, 2.010, 2.006) has been proposed to have similarity to that of the proximal [3Fe4S] cluster in [NiFe]-hydrogenase, which has a principal *g* values of 2.03, 2.01, and 2.00,^52^ suggesting a similarity in the mechanism of oxygen stability between [FeFe] and [NiFe] hydrogenases, although this hypothesis has been challenged.^28^ Our data show that this mechanism is not relevant to *Cb*A5H, as the proximal [4Fe4S] cluster displays standard cubane geometry, and the extra cysteine does not participate in cluster coordination.

The role of the Fd domains in oxygen stability of [FeFe]-hydrogenases has previously been debated.^36–38^ Removal of the Fd domains from *Me*HydA did not affect its relative oxygen stability, as this is likely mediated by restricted gas channels that hinder the access of oxygen to the active site,^38^ rather than the binding of sulfur to the metal center, which is the case in *Cb*A5H.

However, sequence alignment demonstrates that the Fd domain of oxygen-protected [FeFe]-hydrogenases (*Cn*HydA1, *Cb*A5H, *Dd*H) is smaller and features polar residues around the iron sulfur clusters, when compared to oxygen-sensitive hydrogenases (e.g., *Cp*I) (Figure S11). Our results show a decrease in the oxygen stability of *Cb*A5H variants C236A, H245A, and C236A/H245A, reflected by a significant decrease in specific activity after oxygen exposure relative to the WT. It was previously reported that the C236A variant had no effect on oxygen stability of *Cb*A5H,^47^ however, the variant was not characterized in detail as we report here. Our FTIR characterization shows that all variants retain the same redox state signature and the ability to form H_inact_, ruling out the possibility of attributing the decrease in oxygen stability to the direct effect of the mutations on the H-cluster structure. However, the FTIR data also show that the C236A and C236A/H245A, in contrast to the WT, are unable to completely form H_inact_ after 30 min of air exposure. Cyclic voltammograms demonstrate that the C236A variant’s hydrogen oxidation peak has shifted to a slightly more positive potential compared to the WT, and notably, kinetic investigations by potential step chronoamperometry indicated decreased rate constants for inactivation. We therefore attribute the loss of oxygen stability in *Cb*A5H C236A to the slower formation of H_inact_.

Recent efforts in characterizing group B [FeFe]-hydrogenases have found that enzymes with three cysteines in their active site binding motif form H_inact_. However, not all H_inact_-forming enzymes were oxygen-stable. *Cp*III forms H_inact_,^22,53^ but it is not oxygen stable.^24^ Fasano et al. suggested that the reason for oxygen sensitivity despite being able to form H_inact_ is likely due to the slow formation of that state.^23^

Likewise, despite *Cb*A5H C236A preserving the ability to form H_inact_, its oxygen stability decreases because of a slower rate of forming H_inact_. Therefore, we propose that the ability to form H_inact_ is not enough for oxygen stability in *Cb*A5H, and also, the rapid formation of this state, among other reasons, is yet to be explored. Nevertheless, we cannot distinguish whether C236 is affecting the rate of forming H_inact_ due to a change in the midpoint potential or electron transfer kinetics of the proximal iron–sulfur cluster in the Fd domain. Either could influence the oxidation rate of the H-cluster and, therefore, the rate of forming H_inact_ and oxygen stability of the enzyme.

Chronoamperometry of *Cb*A5H H245A showed an increased rate of H_inact_ formation, supporting the observations from FTIR. We therefore attribute the loss of oxygen stability in that variant to another mechanism. It is possible that, being closer to the surface of the protein, replacement of H245 with a smaller side chain enhances oxygen diffusion to the distal metal cluster, interfering with the electron transfer pathway. It is worth noting that *Cb*A5H’s H245 appears to be conserved in *Dd*H (H58), another oxygen-stable [FeFe]-hydrogenase (Figure S11). Inspecting the crystal structure of *Dd*H (PDB: 6SG2) shows that H58 is 3.7 Å away from the distal iron sulfur cluster (Figure S27). This striking similarity between these two oxygen-stable [FeFe]-hydrogenases hints that this residue may be important for oxygen stability in *Dd*H as well. The *Cb*A5H C236A/H245A shows kinetic features of both individual mutations, but does not display significant synergy.

*Cn*HydA1, a novel enzyme that is as oxygen stable as *Cb*A5H, served as an informative agent for comparison. *Cn*HydA1 displays differences in residues that have been previously associated with the oxygen stability in [FeFe]-hydrogenases. *Cb*A5H’s A561 was proposed, along with L364 and P386, to be crucial for conferring oxygen stability, but this residue is not conserved in *Cn*HydA1, being substituted by a much larger leucine residue (Figures 6B and S11), suggesting that the three residues may not have to be strictly conserved for the loop flexibility to occur. In addition, *Cb*A5H’s M382 is substituted by leucine in *Cn*HydA1, demonstrating that while this may influence oxygen stability by controlling protein flexibility, it is not essential.

In contrast to *Cb*A5H, *Cn*HydA1 lacks an SLBB domain. This domain is found in membrane-bound proteins, polysaccharide export proteins, and the animal vitamin B12 uptake proteins, yet it is seldom found in [FeFe]-hydrogenases. Previous attempts to truncate the SLBB domain of *Cb*A5H showed that the enzyme lost all activity, preventing a detailed investigation into the role of this domain in oxygen stability.^47^ Characterizing *Cn*HydA1, a naturally occurring oxygen-stable [FeFe]-hydrogenase that lacks the SLBB domain, indicates that the SLBB domain is likely not required for oxygen stability in *Cb*A5H. More recently, it was shown that this domain is relevant for dimerization and protein stability in *Cb*A5H.^25^

## Conclusion

In conclusion, we provide a high-resolution X-ray structure of *Cb*A5H, where we clarify the identity of the ligand required to confer protection from oxygen attack. We characterize a novel [FeFe]-hydrogenase *Cn*HydA1, which lacks the SLBB domain, and we use it as evidence to suggest that the SLBB may not be required for *Cb*A5H oxygen stability. We show that the Fd domain of *Cb*A5H features polar residues in the vicinity of the proximal and distal iron sulfur clusters, namely, C236 and H245. When these residues are mutated to alanine, *Cb*A5H’s oxygen stability significantly decreases. We show that these variants alter the inactivation and reactivation kinetics of *Cb*A5H. In the C236A variant, we attribute the decrease in oxygen stability to the slower rate of H_inact_ formation. Future studies that combine the accurate estimation of the midpoint potential of the accessory iron sulfur clusters will be instrumental in determining their role in oxygen stability of [FeFe]-hydrogenases. Our results provide a more detailed understanding of oxygen stability mechanisms in *Cb*A5H-like [FeFe]-hydrogenases and inform future research aimed at producing more stable biocatalysts for sustainable H_2_ technologies.

## Supplementary Material



## Notes

Materials and methods; list of primers and plasmids; crystallographic statistics; sequence identity table of *Cn*HydA1, *Cb*A5H, and *Cp*I; table of H_ox_ and H_inact_ FTIR profile of *Cb*A5H, *Cn*HydA1, *Dd*H, *Cp*III, and *To*HydA; table showing the specific activity of the enzymes presented in the main text anaerobic and after air exposure, including *Cb*A5H WT, *Cb*A5H C236A, *Cb*A5H H245A, *Cb*A5H C236A/H245A, *Ca*HydA1, and *Cn*HydA1; density maps, anomalous maps, protein model illustrations, including the proton transfer pathways for *Cb*A5H and *Cp*I and *Dd*H and hydrogen bonding network in *Cb*A5H; multiple sequence analysis and structural alignment of *Cb*A5H and *Cp*I Fd domains; cyclic voltammetry measurements of *Cb*A5H and its variants at pH 8.0 and 100% H_2_ atmosphere, at pH 8.0 and 100% N_2_ atmosphere, and at pH 6.0 and 100% H_2_ atmosphere; gas-saturated buffer injection electrochemistry experiments; potential-time sequence used to probe *Cb*A5H anaerobic inactivation; summary of *Cb*A5H and variants baseline-corrected, averaged, normalized current response, and simulated current response to the potential step experiments; simulation parameters; phylogenetic distance scheme of *Cn*HydA1 to *Cb*A5H. SDS-PAGE of *Cn*HydA1 after purification; FTIR spectra of *Cn*HydA1 when exposed to CO and when purified aerobically; figure showing the distance of H58 to the distal iron sulfur cluster in *Dd*H; additional references are cited in the Supporting Information^8,54–62^ (PDF)
