# Supplementary material for: Probing the Role of Accessory Domains in Oxygen Stability of [FeFe]-Hydrogenases
Source: J Am Chem Soc. 2026 Jul 10;148(28):29985–96. doi: 10.1021/jacs.6c05865 (PMC13397568; doi:10.1021/jacs.6c05865)
Supplement: Supplementary file 1 [file ja6c05865_si_001.pdf]

Supporting Information for

Probing the role of accessory domains in oxygen stability of [FeFe]-hydrogenases

Abdulrahman Alogaidi[a], Stephen Carr[b,c], Lucy Hudson [d], Henry Lloyd-Laney[d], Alison Parkin[d], Ashley Love[e], Michael W. George[e], Anca Pordea[a], Simone Morra[a*]

[a]: Faculty of Engineering, University of Nottingham, University Park, Nottingham NG7 2RD, United Kingdom.

[b]: Department of Chemistry, University of Oxford, Oxford OX1 3QR, United Kingdom.

[c]: Research Complex at Harwell, Rutherford Appleton Laboratory, Didcot, Oxfordshire OX11 0FA, United Kingdom.

[d]: Department of Chemistry, University of York, Heslington, York, YO10 5DD, United Kingdom.

[e]: School of Chemistry, University of Nottingham, University Park, Nottingham NG7 2RD, United Kingdom

Materials and Methods

In silico analysis

Primary structure alignment, structure prediction and visualization, genomic sequences and DNA suppliers

Amino acid sequences accession numbers are WP_011721785.1 for *Cn*HydA1, WP_026887313.1 for *Cb*A5H, WP_004455619.1 for *Cp*I, WP_010939057.1 for *Dd*H, and WP_010963357.1 for *Ca*HydA1. Sequence alignments were computed in Clustal Omega [1], and phylogenetic analysis was performed using MEGA11 software [2].

The genomic sequence of *Clostridium novyi* strain NT was taken from the N*CB*I database (accession ID NC_008593.1) and the gene accession number of *Cn*HydA1 is NT01CX_RS04100.

Cloning

Primers (*Table S 2*) were sourced from Integrated DNA Technologies (Belgium). Genomic DNA samples were sourced from Dr Ian Cheong (*C. novyi* NT).

Genes were amplified using PCR from the genomic DNA using Phusion™ High-Fidelity DNA Polymerase sourced from New England Biolabs, and assembled with pET plasmid modified with a N-terminal Twin-Strep-Tag, followed by a TEV cleavage site using Gibson assembly [3].

Plasmid were assembled with NEBuilder® HiFi DNA Assembly (New England Biolabs) and according to manufacturer instructions. The plasmids were extracted and purified according to the manufacturer instructions (QIAprep Spin Miniprep Kit | Plasmid DNA Isolation). Plasmids were sequenced to confirmed correct assembly using Sanger sequencing (Eurofins genomics services).

Site directed mutagenesis

Primers for mutagenesis were designed using quick change online tool by Agilent technologies. KOD polymerase hot start (Merck, Germany) was used in the reaction. The reaction mixture included, in a final volume of 20 µl, template plasmid containing the hydrogenase gene 2ng/µl, forward and reverse mutagenesis primers (final concentration of 0.25 µM), dNTP (final concentration of 0.2 mM), reaction buffer (final concentration of 1x) and 0.4 µl KOD polymerase. The PCR cycle was the same for all mutagenesis reactions, which is 95 °C for 2 minutes, then 25 cycles (95 °C for 1 minute, 55 °C for 1 minute, then 70 °C for 25 s/kb), then final extension for 5 minutes at 70 °C. The reaction mixture was then digested with DpnI for the removal of template DNA (1.5 µl of FastDigest DpnI (Thermo FD1703) in the 20 µl reaction mix; incubated for 2 hours at 37 °C). 5 µl of the product was used to transform competent cells. Plasmids were sequenced to confirmed correct mutagenesis using Sanger sequencing Eurofins genomics services.

Hydrogenase Expression

*Cb*A5H WT, C236A, H246A, C236A/H245A and *Cn*HydA1 were coexpressed with maturases *Ca*HydEFG in *E. coli* BL21(DE3) (ΔiscR) [4]. Cells were grown in terrific broth (TB), as a preculture of 50 ml, supplemented with ampicillin (100 µg/ml), streptomycin (50 µg/ml), kanamycin (30 µg/ml) overnight at 37°C with 200 rpm shaking. The pre culture was used to inoculate 0.5 L TB media to a starting OD_{600} of 0.05 supplemented with ampicillin (100 µg/ml), streptomycin (50 µg/mL), kanamycin (30 µg/ml), ammonium ferric citrate (final

concentration of 0.5 mM) and cells were grown to OD of 0.6-0.8. Protein expression was induced by addition of IPTG to a final concentration of 0.5 mM, with the addition of 2 mM cysteine, 0.5% glucose, 25 mM sodium fumarate (final concentrations), and incubated overnight at 18 °C with nitrogen sparging in an anaerobic bottle [5].

Hydrogenase Purification

Cells were collected by centrifugation (6000g for 20 minutes) and stored at -20 °C until required. Pellet was lysed under anaerobic conditions using lysis buffer (100 mM Tris HCl, 150 mM NaCl, pH 8, 0.5% Triton X-100, 5% glycerol,1 cOmplete protease inhibitor tablet, 1mg/ml lysozyme, and benzonase 1µl/10ml (stock concentration of ≥250 units/µl). After 1 hour of stirring DTT was added to final concentration of 5 mM. Cells were stirred for additional 30 minutes and centrifuged for 20 minutes at 22,500 rpm at 10 ˚C. Cell lysates were purified using Cytiva 5 ml StrepTrap HP or IBA 5 ml StrepTactin Superflow high capacity cartridges. The column was washed with buffer 100 mM Tris HCl, 150 mM NaCl, pH 8 with three column sizes, and protein was then eluted with 5 mM desthiobiotin [6]. Protein concentrations were estimated using Bradford assay.

For anaerobic purification of the samples, all the procedure described above was done in the anaerobic chamber (Whitley A85 Anaerobic Workstation). Buffers used were stirred in the chamber for a minimum of 1 hour.

For crystallisation of *Cb*A5H, cells were harvested aerobically and the whole purification process was done aerobically. After a first step of affinity chromatography, TwinStrepTag was removed using TEV protease with 1:20 mass to mass ration of TEV to *Cb*A5H. The digestion reaction was carried for 1 hour at 30 °C with 150 rpm shaking. The protein was then further purified from the undigested residues and TEV protease using the affinity chromatography StrepTrap. Tag-free protein was then further purified with Size exclusion chromatography, and fractions corresponding to the dimer (molecular 143.6 kDa) were pooled and concentrated to 25 mg/ml using Vivaspin from Sigma-Aldrich.

TEV purification

TEV was produced with an N-terminal StrepTagII sequence for ease of removal post digestion. pRK793-Strep (available from Addgene #240334) has been generated by replacing the His_6-tag in the parent pRK793 (Addgene #7792) with a StrepTagII sequence. pRK793-Strep was transformed in *E. coli* Rosetta2(DE3) and the enzyme was expressed and purified as previously described [7], except that affinity chromatography was done on Cytiva 5 ml StrepTrap HP or IBA 5 ml StrepTactin Superflow high capacity cartridges.

*Cb*A5H crystallization, data collection and processing

Crystallisation conditions were identified by screening the protein against several commercially available crystallisation screens (JCSG, Morpheus, PGA, and SG1 from Molecular Dimensions Ltd, UK). Crystals were grown using the vapour diffusion method by mixing 100 nl protein solution (25 mg/ml) with an equal volume of crystallisation buffer using a mosquito robot (STP Labtech, UK) and equilibrating against a 45 µl reservoir. After incubation for 2-3 days at 293K crystals were observed in two conditions: (1) 0.2 M $MgCl_2$, 0.1 M HEPES 7.5 pH, 30 % PEG 400, (2) 30 %w/v PEG (PEG 8000 and ethylene glycol 1:2 ratio), 0.1 M HEPES and MOPS 7.5 pH (Buffer), and 0.1 M MCA (Sodium formate, Ammonium acetate, Sodium citrate, tribasic dihydrate, Potassium sodium tartrate, and Sodium oxamate, with each acid constituting 0.02 M). After appearing, crystals continued to grow for up to a week. The first condition was further optimized to 34% PEG 400, 0.2 M

$MgCl_2$ and 0.1M HEPES pH 7.3. Crystals were collected and cryocooled in liquid nitrogen prior to x-ray data collection.

Data sets were collected at Beamline I03 (Diamond Light Source, UK) using x-rays with a wavelength of 0.97 Å. X-ray data reduction was performed automatically using XIA2 [8] and DIALS [9]. Initial phase estimates were calculated using PHASER [10] with PDB: 6TTL as a search model. Model building and manual refinement was carried out using COOT [11] and model was further refined using Phenix refine. Rebuilding and refinement were performed iteratively until no further improvements were possible. Final structure has been deposited in the PDB with accession code 9RKM.

Activity in hydrogen evolution

The activity of the enzyme in hydrogen evolution was estimated using GC-TCD. The enzyme was reacted in 2ml (100 mM TrisHCl, 150 mM NaCl, pH8, 10mM methyl viologen, 20 mM sodium dithionite) in 20 ml enclosed vials, sparged with Argon and incubated 20 minutes at 37 °C. 5 µl of the enzyme stock solution (0.2 mg/ml) was then injected and the vial was incubated for another 20 minutes at 37 °C. A 50 µl sample of the headspace gas of the vial was taken and measured, and the area under peak corresponding to the retention time of hydrogen was used to estimate the total volume of hydrogen produced through a standard curve constructed with known volumes of hydrogen. Three replicas per experiments were used to estimate the hydrogen production, as reported by [6]. Significant differences were calculated using T test with two tail distribution.

FTIR spectroscopy

The enzyme was concentrated to 1-2 mM and placed in a FTIR transmission cell (PIKE Technologies, 10 µl), equipped with CaF_2 windows and 50 µm Teflon spacer. Spectra were acquired (Thermo Fisher Scientific Inc.Nicolet iS50R, equipped with a liquid N_2 cooled TRS MCT detector, with a spectral resolution of 2 cm^{-1}, accumulating 1024 scans). The final IR spectra of the sample was obtained by recording a spectrum of the buffer containing the enzyme against an air background, with a buffer against air background control spectrum subtracted using the OPUS software. The baseline was further corrected using Origin Pro 2024.

Protein Film Electrochemistry

All electrochemical measurements were undertaken in a glovebox supplied with a N_2 atmosphere (O_2 < 5 ppm). The electrochemical cell was jacketed, allowing cooling to 5 °C via a thermostatically controlled water bath. The cell was gas tight, gas was supplied via a needle and septum, and a bubbler outlet was used to ensure equilibration to atmospheric pressure. Unless otherwise stated, the buffer used was 5 mM of each of HEPES, sodium acetate, TAPS, CHES and MES with 140 mM NaCl and the pH was adjusted using conc. HCl and NaOH. Buffer solutions were purged with 100 % N_2 before storing in the glovebox.

The electrochemical cell components comprised a Pt wire counter electrode, Ag/AgCl reference electrode and OrigaTrod – Rotating Disk Electrode with a pyrolytic graphite edge disk as the working electrode. All measurements were run using a Ivium CompactStat2.h and recorded using IviumSoft. The reference electrode was converted to SHE using the onset-potential values of the *Cb*A5H WT measured for that experiment.

Working electrodes (WE) were polished with aluminium slurry outside of the glovebox. Electrodes were then polished with sandpaper inside the glovebox before applying a protein film by pipetting 1 µl onto the WE surface and allowing the films to dry under N_2. The

enzyme solutions used consisted of *Cb*A5H WT (1.8 mg/ml); *Cb*A5H C236A (1 mg/ml); *Cb*A5H H245A (1 mg/ml); *Cb*A5H C236A/H245A (3 mg/ml), all enzyme aliquots were stored in 100 mM Tris HCl, 150 mM NaCl, and pH 8 buffer.

Cyclic voltammetry measurements were taken under the conditions stated in the figure caption. A scan rate of 3 mVs^{-1} and an E_{step} of 2 mV were used. Cyclic voltammograms were also measured before undertaking any chronoamperometry measurement to confirm an adequate protein film. The details of the potential step experiments are provided in Figure S 19 and Table S8.

Enzyme inactivation model

Potential-induced enzyme inactivation and reactivation of *Cb*A5H under an atmosphere of 100% H_2 at pH 8.0, 5 °C is modelled using the reaction scheme published by Winkler et al[12] which assumes two different active states of the enzyme, A_1 and A_2, reversibly inactivate to state I (Figure S 1). At the same time, there is also irreversible loss of enzyme from the experiment, accounted for by formation of a lost state, L.

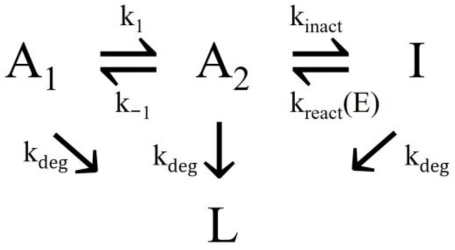

Figure S 1. Reaction scheme to account for reversible anaerobic inactivation of Cb A5H

The total amount of enzyme is assumed to stay constant across the experiment, as summarised in Equation 1.

$$1 = [A_1]_t + [A_2]_t + [I]_t + [L]_t$$

Equation 1

The evolution of the relative proportions of the system can be modelled as a system of ordinary differential equations, assuming mass action kinetics, such that:

dA1/dt=k_-1[A2]-(k_1+k_deg)[A1]

dA2/dt=k_1[A1]+k_react(E)[I]-(k_inact+k_-1+k_deg)[A2]

dI_dt=k_inact[A2]-(k_react(E)+k_deg)[I]

Given initial values for each of [A1], [A2] and [I] and values for each of the kinetic parameters, estimates for the concentrations of each of the three species as a function of time and potential can be estimated using numerical simulation.

However, the chronoamperometry data is a single current trace, which is a linear sum of the Faradaic current (i_f), arising from the electrocatalytic oxidation of hydrogen, and the capacitative current (i_c) that arises from non-electron transfer processes occurring on the surface of the electrode. These are generally ascribed to changes in the double layer upon a change in the electrode potential.

The challenge is therefore relating the relative proportions of the species in the ODE model to the total current. The approach we have selected is as follows

$i\_f(t) = Q(2[A1](t) + [A2](t))$

where Q is a scalar, with the relative contributions of A1 and A2 following the observation that species A1 has twice the activity of species A2 by Winkler et. al. Ideally, we would wish to obtain an estimate for the current observed based on the surface coverage, k_cat of the enzyme, number of electrons transferred and Faraday's constant, rather than an unconstrained scalar. Unfortunately, there is no way to estimate the surface coverage of the enzyme from chronoamperometry data alone. Winkler et al . used background subtraction to obtain a purely Faradaic current. We take a slightly different approach,

$i\_tot(t)=i\_f(t)+C\ i\_b(t)$

such that the total current i_tot(t) is equal to the sum of the Faradaic current and the recorded background current multiplied by a scalar C. We can then use the objective function

RMSE(i_tot_exp, i_tot_simulation)

to obtain estimates for the kinetic parameters k_1, k_-1, k_inact, k_react, the initial proportions of each of the three species and the scaling factors Q and C as part of an optimisation, where these parameters are systematically altered to determine the values that minimise the distance between i_tot_exp and i_tot_sim. This approach allows us to determine the extent to which the scaling factors Q and C (analogous to the choices made during normalisation and background subtraction) affect the estimates of the kinetic parameters.

The nature of the chronoamperometry data means there are several choices that must be made before running an optimisation.

1) Data to be included in the optimisation
    a) At the most constrained level, this means that the total chronoamperometry experiment is included as part of a single optimisation, with the initial proportions of A_1, A_2 and I only set at t =0.
    b) At the least constrained level, every individual change in potential can be modelled as a separate experiment, with the kinetics, conversion values and initial conditions being optimised separately.
    c) There are a large number of combinations between these two extremes. A balance has to be struck between getting a good fit to the experimental data, and recovering parameters that can tell us something meaningful about the kinetics of the hydrogenase active site inactivation and reactivation
2) Capacitive "spikes": after a change in potential, there is a large spike in current, particularly after the transition from the -0.8 V potential pulse to more positive potentials. The current magnitude of these spikes is significantly larger than the current that occurs subsequently, and as such these features can dominate the objective function. This is problematic, because these spikes do not provide much information about the kinetics of hydrogenase. As such, it is possible to exclude currents for a set period time after potential step changes.

3) Bounds and “fixing” parameters: the region of parameter space in which an optimiser searches is pre-set (or a particular value can be fixed *a priori).* Certain regions which are judged to be unphysical can be excluded for this reason.

All optimisations were run twenty times independently, starting from a random initial position within the bound for each parameter. The optimiser (the CMA-ES algorithm, implemented in the PINTS repository) was run until 200 iterations had passed without an improvement in the objective function score below a threshold of 1e-5 amps. We have additionally used Markov chain Monte Carlo approaches to explore the region of parameter space around the CMA-ES recovered parameters for some optimisation approaches. In this case, two chains were initialised from the CMA-ES value and run for 10000 iterations.

In Figure S2, we show the effect of varying the individual parameters of the model while holding the others constant. We can see first that Q and C have their expected effects, acting as simple scalars of the Faradaic and capacitative current respectively. In general, all four kinetic parameters largely affect the current value of the steady state, and the rate at which the current converges to this steady state, which we term the transient (although more complex oscillating dynamics can be observed with some combinations of k_1 and k_{-1}). This serves to illustrate the use of excluding the capacitative spikes from the objective function. Determining the kinetic behaviour of the system requires accurately determining the steady state current after a potential transition, and the transient before this steady state.

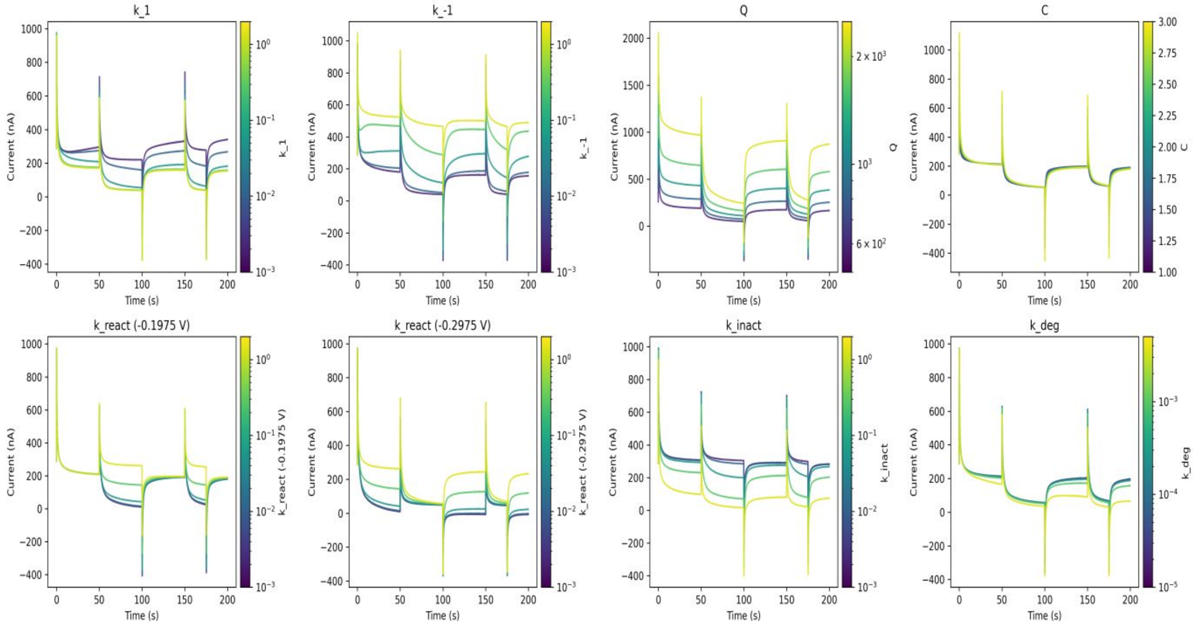

Figure S 2. Parameter scan showing the effect of varying one parameter (title and colourbar) when every other value is held constant.

With this in mind, we compare three model combinations (approaches 1-3 in Figure S3) in their ability to capture the dynamics of a low potential and high potential pulse for the wild-type enzyme; raw data is shown in figures later in the SI. In all approaches, the initial capacitative spike for 5 seconds after a potential transition was not included in the objective function. In approach 1, each individual pulse transition has been separately optimised using different kinetic, current conversion values and initial proportions of A_1, A_2 and I. In approach 2, each “chunk” (i.e. the sequence of potentials in between the -0.54 V pulses as shown in

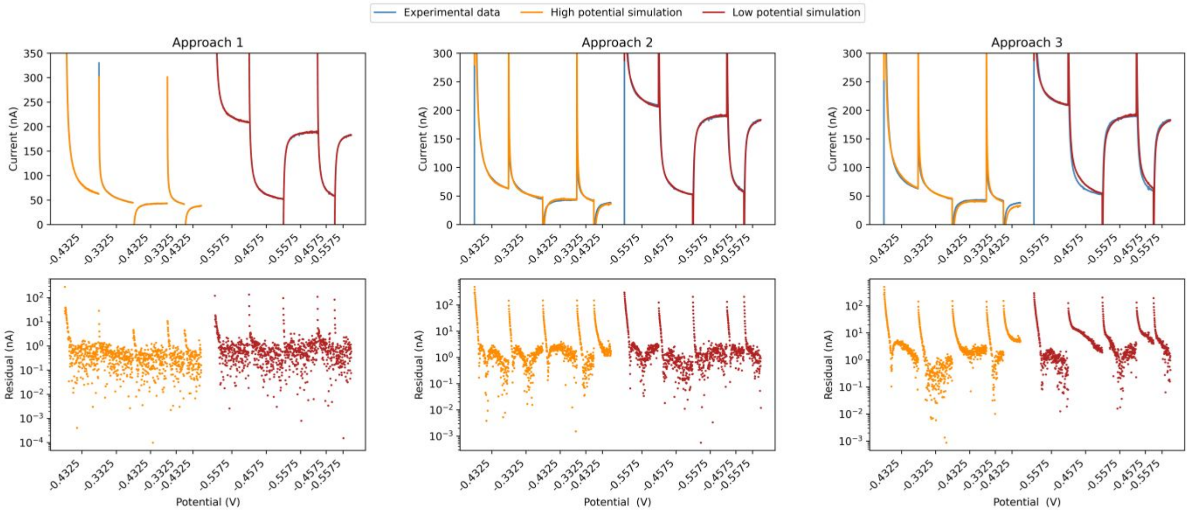

Figure S 3. Comparison between experimental WT data (blue) and simulation for a phase at "high" potentials (orange) and "low" potentials (red) for three different modelling approaches. Residuals for the total phase are shown in the lower row.

Figure S19) has been optimised separately, with initial proportions of A_1 and A_2 set after the first transition out of -0.54 V (following Winkler et al. the initial concentration of I was set to 0). k_{inact}, k_{deg}, k_1 and k_{-1} were kept at the same value throughout the "phase" simulation, and k_{react} was allowed to take a different value for each distinct potential. This approach follows Winkler et al.'s observation that k_{react} has a dependence on potential, but imposes no constraint as to the functional form of this relationship. Each phase was then optimised separately.

In approach 3, the Q and C values for each "phase" were pre-specified at the values determined from approach 2. Each "phase" was simulated separately with the initial proportions at the start of each step up from H_2 production conditions given individual parameters in the optimisation. The values of k_{inact}, k_{deg}, k_1 and k_{-1} were kept the same across all stages, and k_{react} allowed to vary with potential, as in approach 2 (if two pulses in different H_2 oxidation stages were at the same potential value, then the value of k_{react} would be identical for these two pulses). The objective functions for each phase were calculated separately and then summed to be passed to the optimiser.

These approaches can be loosely grouped in order of constraint. Approach 1 is maximally unconstrained, with no information between potential pulses shared. For example, the final species proportions prior to a potential transition are not taken into account when determining the initial conditions after the transition. In approach 2, each H_2 oxidation phase is independent, but initial proportions are only set at the start of each phase and not with each potential transition. Finally in approach 3, the optimiser must seek to provide a good fit to all phases simultaneously, albeit with Q and C parameters pre-defined separately for each phase. This latter modelling choice was implemented after observing the results from objective 2, where A) there was a high level of consensus as to the value of Q and C between high-scoring parameter vectors and B) these values increased in phases that were recorded later in the experiment. As both Q and to a lesser extent C indirectly report on the unmeasurable surface coverage value, this trend indicates a progressive reduction in the surface coverage. To avoid simultaneously optimising Q and C separately for each phase (requiring an extra 30 parameters in the optimisation), or requiring a single common value, we compromised by pre-specifying the value separately for each phase.

As would be expected, as the modelling approach moves from less to more constrained, the quality of the fit between simulation and data degrades. Approach 1 can describe the steady

state and transient extremely well. In the lower row of Figure S3, we show the residual without excluding the capacitive spikes. The very large errors in these regions (two-three orders of magnitude larger than in the steady state) reinforce our decision to exclude this feature from the optimisation. For approach 1, the residuals indicate that the deviation between simulation and data is largely un-systematic (i.e. primarily driven by noise). In approach 3, the most constrained version, we can see that the model is systematically failing to capture both the current steady-state and the dynamics of the transient. Approach 2 does a much better job at capturing these dynamics, although it still has more systematic error than approach 1. Even more constrained approaches (for example, optimising the whole experiment without splitting into phases and a single value for each parameter) yielded even worse fits.

Our ultimate solution was to settle on approach 2. This is because approach 1 is both mechanistically unsatisfying, in that the relative proportion of A_1, A_2 and I is not conserved even over a H_2 oxidation phase, and resulted in a very large spread of plausible parameter values. We show this in Figure S4 below, comparing best-fit parameter values within 5% of the best scoring vector recovered from analysing all repeats and potential phases in the WT dataset. We should note that, after exploring the results from approach 1 further, we concluded that kinetics values over $1s^{-1}$ tended to instantly equilibrate to the steady state, and that in this kinetics regime all dynamics were modelled by the combination of background current and degradation. As this is unlikely (it renders the two-state model proposed by Winkler et al. moot, for one), in approach 2 we restricted the parameter bounds to a maximum of $1s^{-1}$.

Having settled on approach 2, we now wish to analyse the parameters recovered by the optimisation efforts. We have explored parameter space in some detail. Parameters for each variant and the wild type (4 total), H_2 oxidation phase (15 total) and repeat (3 total) were optimised 20 times, resulting in 3600 parameter sets. The results are summarised in Table S1. The "Mean" column refers to the average of the best fit values for each phase. The "Per repeat" column shows the 10th-90th percentile of the recovered best values across each repeat and H_2 oxidation phase. The "Per potential" column shows the same variation across the potentials, but only for repeat 1. Per high score shows the range of values across all H_2 oxidation phases and repeats and also includes every set of parameter values which yield a score within 5% of the best value for each phase. What we can see here is that a large spread of values can be associated with a "good" fit, which is presumably driven by the degrees of freedom of the model. This is particularly acute in the cases of the parameters of Q and C, which are phenomenological and cannot be interpreted directly as physical quantities.

Table S 1. Table showing collated results from all optimisations, across all repeats, variants and potential phases.

Variant	Parameter	Mean	Per repeat	Per potential	Per high score
WT	$[A_1]$	0.45	[0.08, 0.97]	[0.12, 0.98]	[0.21, 0.95]
WT	k_{inact}	0.38	[0.22, 0.76]	[0.31, 0.71]	[0.32, 0.99]
WT	k_{deg}	0.00107	[3.84e-04, 2.96e-03]	[3.91e-04, 2.90e-03]	[8.70e-04, 3.65e-03]
WT	Q	456.24	[175.65, 1546.46]	[147.16, 922.81]	[149.89, 396.5]
WT	C	2.1	[1.66, 4.1]	[1.58, 2.51]	[1.5, 2.51]

WT	k_1	0.08	[0.03, 0.12]	[0.05, 0.13]	[0.09, 0.16]
WT	k_{-1}	0.01	[1.00e-05, 0.03]	[1.00e-05, 0.04]	[1.00e-05, 0.21]
WT	k_{react}	0.16	[0.03, 0.31]	[0.06, 0.28]	[0.06, 0.71]
C236A	$[A_1]$	0.56	[0.08, 0.99]	[0.08, 0.99]	[0.13, 0.99]
C236A	k_{inact}	0.34	[0.08, 0.59]	[0.1, 0.99]	[0.14, 0.99]
C236A	k_{deg}	0.000979	[4.83e-04, 1.87e-03]	[5.50e-04, 1.62e-03]	[5.20e-04, 1.88e-03]
C236A	Q	768.9	[211.38, 1016.15]	[184.38, 1523.66]	[193.49, 827.17]
C236A	C	2.06	[1.72, 4.75]	[1.47, 3.37]	[2.05, 3.96]
C236A	k_1	0.05	[0.03, 0.61]	[0.04, 0.06]	[0.04, 0.83]
C236A	k_{-1}	0.02	[1.52e-05, 0.03]	[6.82e-03, 0.03]	[3.07e-03, 0.3]
C236A	k_{react}	0.18	[0.03, 0.44]	[0.03, 0.22]	[0.06, 0.88]
H245A	$[A_1]$	0.63	[0.12, 1.0]	[0.23, 1.0]	[0.19, 1.0]
H245A	k_{inact}	0.72	[0.32, 1.74]	[0.38, 1.5]	[0.41, 1.96]
H245A	k_{deg}	0.00196	[5.47e-04, 4.96e-03]	[6.01e-04, 4.88e-03]	[7.46e-04, 5.00e-03]
H245A	Q	663.15	[287.95, 1816.62]	[279.48, 894.82]	[276.77, 893.67]
H245A	C	2.95	[1.88, 6.47]	[2.08, 3.83]	[2.14, 3.85]
H245A	k_1	0.09	[0.05, 0.13]	[0.05, 0.11]	[0.05, 0.12]
H245A	k_{-1}	0.06	[3.15e-04, 0.2]	[1.00e-05, 0.03]	[1.00e-05, 0.03]
H245A	k_{react}	0.31	[0.03, 0.72]	[0.05, 0.65]	[0.09, 1.32]
C236A/ H245A	$[A_1]$	0.57	[0.11, 1.0]	[0.08, 1.0]	[0.13, 1.0]
C236A/ H245A	k_{inact}	0.39	[0.16, 1.31]	[0.23, 1.01]	[0.31, 1.98]
C236A/ H245A	k_{deg}	0.00188	[5.02e-04, 4.54e-03]	[5.10e-04, 3.50e-03]	[8.96e-04, 4.35e-03]
C236A/ H245A	Q	504.98	[180.24, 729.5]	[184.23, 431.43]	[192.13, 551.36]
C236A/ H245A	C	2.08	[1.59, 3.25]	[1.63, 2.64]	[1.95, 2.74]
C236A/ H245A	k_1	0.12	[0.04, 0.39]	[0.05, 0.31]	[0.06, 0.15]
C236A/	k_{-1}	0.24	[3.39e-03, 1.12]	[1.08e-05, 1.05]	[1.00e-05, 0.98]

H245A					
C236A/ H245A	k_{react}	0.16	[0.01, 0.43]	[0.02, 0.44]	[0.03, 1.32]

Finally, to decompose some of these sources of variation, we performed MCMC, as detailed above. After initial investigations, the chains did not converge when every parameter in the model was included. As such, we pre-specified the values of Q, C and k_{deg} for each phase to the best fit value determined previously in the optimisation attempt. After this the chains converged, with all R_{hat} values (a measure of convergence) being under the 1.1 threshold specified in the original paper by Gelman and Rubin[13]. Because the MCMC is initialised from the optimum point, the chains are effectively exploring the likelihood surface around the optimum, and if there are many plausible values of Q and C that can describe the data, this will cause non-convergence in the chains. Choosing any particular background subtraction and normalisation approach is analogous to pre-specifying a Q and C value.

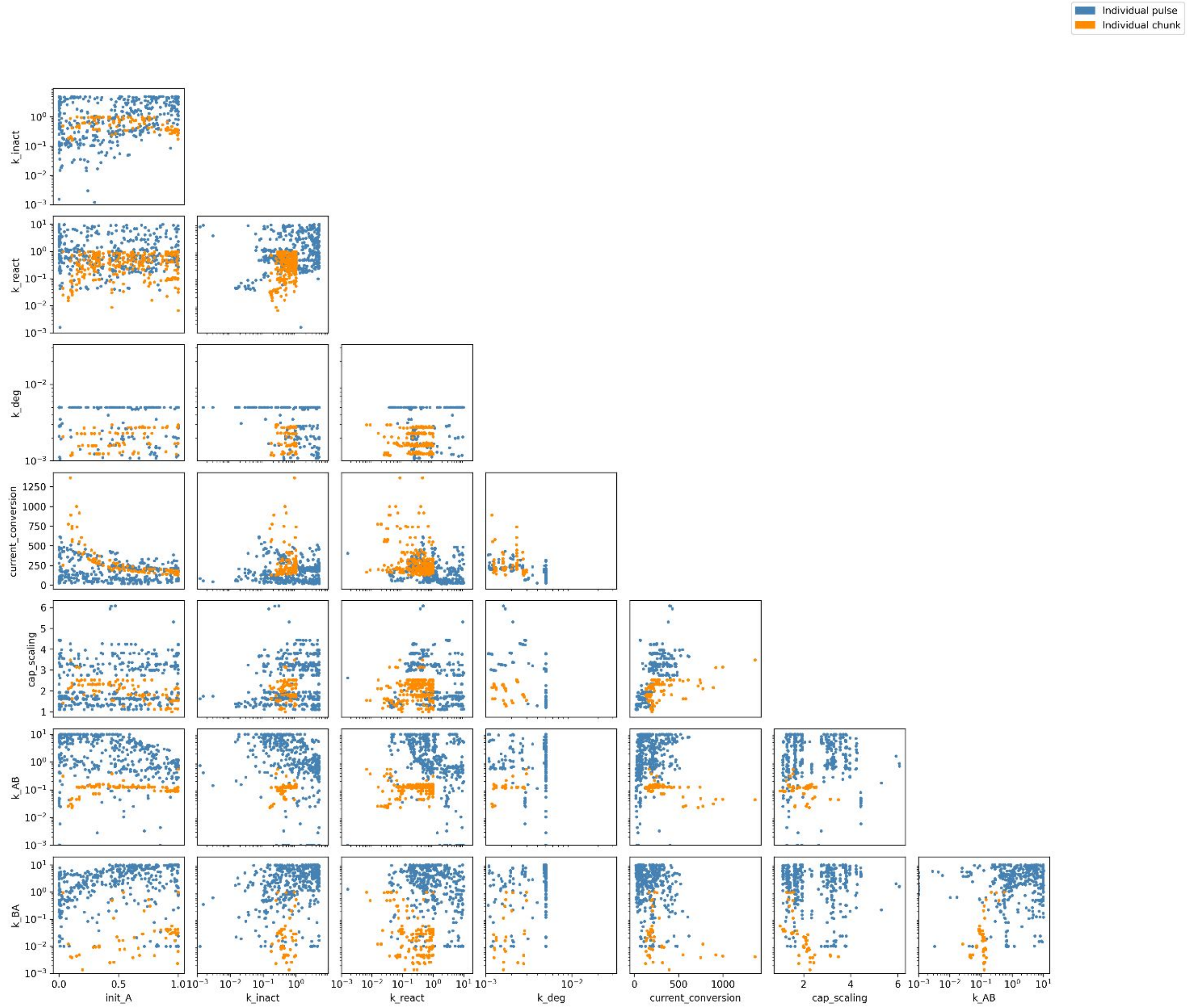

Figure S 4: Two dimensional parameter plots showing parameter values associated with the top-scoring optimisation runs (defined as an objective score within 5% of the optimum for each chunk) for modelling approaches 1 and 2.

In Figure S5, we can see that there is, in general, remarkably good agreement between kinetic parameter estimates between repeats for each H_2 oxidation phase, and that, when Q and C are fixed, there is little uncertainty in the parameters that can be attributed to the agreement between simulation and data. Furthermore, the estimates for the WT values of k_1, k_{-1} and k_{inact} are all very similar to those suggested by Winkler et al., and also do not exhibit potential dependency, as was suggested in that work. We do not observe the same exponential relationship between potential and the value of k_{react}, although this relationship may simply be obscured by the multiple sources of variation present in the parameter plot. In the main text, we have suggested based on the collated results that there is a substantial difference between the WT and C236A values for k_{inact}, and the WT and H245A and C236A/H245A values of k_{react}. We can see in Figure S5 that this is primarily informed by H_2 oxidation phases in the "high" potential region for the latter, and for the "middle" potential region for the former.

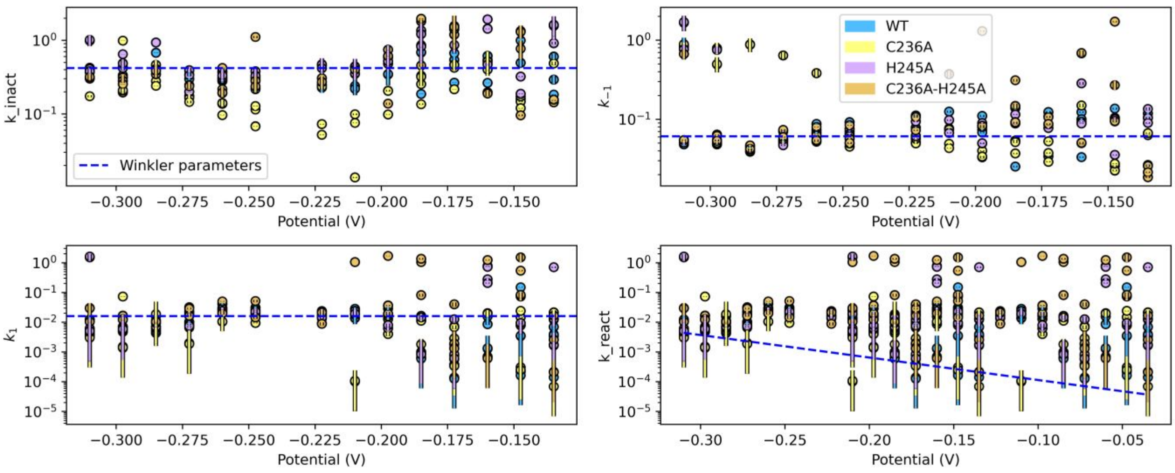

Figure S 5. Mean parameter values (scatter point) for each H_2 oxidation phase, repeat and variant, and associated range of distribution equal to twice the recovered standard deviation in either direction, as recovered by MCMC

Supplementary data

*Table S 2. List of primers used for cloning of Cn**HydA1**, TEV plasmid modification, and Cb**A5H mutagenesis.*

**Cloning**	
*Cn*HydA1 FWD	GAAAATCTGTATTTTCAAGGTGATATTTTTTACTTTCAAATAACAG
*Cn*HydA1 REV	GATCTCAGTGGTGGTGGTGGCTATTTTACATATTCAGTATGAAGATATTTATG
*Cb*A5H FWD	AAAATCTGTATTTTCAAGGTGATAATAAAAAATCTTTTATACAAAGTGC
*Cb*A5H REV	AGTGGTGGTGGTGGTGGTGCTCGAGCTAATCTTTTTTTACTCTTGGG
pET21 backbone FWD	CCACCACCACCACTGAGATC
pET21 backbone REV	ACCTTGAAAATACAGATTTTCGCTG
TEV FWD	TGGAGCCATCCGCAGTTCGAGAAATCCGGAGAAAGCTTGTTTAAGGGGC
TEV REV	CCTGCAGGTCGACTCTAGAGG
**Site directed mutagenesis**	
*Cb*A5H-C236A-FOR	GTAAAAGAGCATGTCCTGTTGATGCCATAAATGGAGAATTAAAAAAGA
*Cb*A5H -C236A-REV	TCTTTTTTAATTCTCCATTTATGGCATCAACAGGACATGCTCTTTTAC
*Cb*A5H-H254A-FOR	TACATCTATTATAATCTATTTCAGCTTTCTTTTTTAATTCTCCATTTATGCAATCAACAGGACA
*Cb*A5H-H254A-REV	TGTCCTGTTGATTGCATAAATGGAGAATTAAAAAAGAAAGCTGAAATAGATTATAATAGATGTA

Table S 3. List of plasmids used.

**Name**	**Relevant characteristics**	**Ref**	**Purpose**
pET21- *Cn*HydA1	T7 Promoter, ampicillin resistance, N-terminal TwinStrepTag, and TEV protease digestion site	This work	For *Cn*HydA1 production
pET21- *Cb*A5H	T7 Promoter, ampicillin resistance, N-terminal TwinStrepTag, and TEV protease digestion site	This work	For *Cb*A5H production
pEFG1	T7 promoter, streptomycin resistance	[14]	For the co-overexpression of maturases HydE, F, G from *Clostridium acetobutylicum*
pRK793-Strep	Derivative of pRK793 with a StrepTagII instead of His_6-tag, now deposited at Addgene #240334	This work	TEV protease to remove the affinity tag of crystallography protein samples

Table S 4. Crystallographic statistics for data collection and structure refinement.

Data set	Native – pdb: 9RKM	Fe anomalous	Zn anomalous
Resolution range	43.66 - 1.96 (2.01 - 1.96)	86.54-3.00 (3.05-3.00)	86.6-2.87 (2.92-2.87)
Space group	P 42 21 2	P 42 21 2	P42 21 2
Unit cell	167.885 167.885 125.679 90 90 90	168.15 168.15 126.20 90 90 90	168.14 168.14 126.47 90 90 90
Wavelength (Å)	0.976	1.734	1.278
Total reflections	6973814 (342464)	2864836 (70650)	3083785 (51683)
Unique reflections	128057 (8978)	36810 (1803)	42034 (2071)
Multiplicity	54.3 (54.4)	77.8 (39.2)	73.4 (25.0)
Completeness (%)	99.86 (99.40)	100 (100)	100 (99.95)
Mean I/sigma(I)	13.3 (0.6)	11.6 (0.5)	14.0 (0.5)
Wilson B-factor	38.62		
R-merge	0.227 (4.796)	0.494 (4.268)	0.368 (3.779)
R-meas	0.229 (4.840)	0.497 (4.324)	0.371 (3.857)
R-pim	0.031 (0.653)	0.056 (0.675)	0.042 (0.768)
CC1/2	0.999 (0.389)	1.000 (0.379)	1.000 (0.345)
CCanom	-	0.7 (0.0)	0.6 (0.0)
Reflections used in refinement	128057 (8978)		
Reflections used for R-free	1910 (136)		
R-work	0.184		
R-free	0.204		
Number of non-hydrogen atoms	10650		
macromolecules	9924		
ligands	154		
solvent	572		
Protein residues	1266		
RMS(bonds)	0.005		
RMS(angles)	0.77		
Ramachandran favored (%)	96.88		
Ramachandran allowed (%)	3.03		
Ramachandran outliers (%)	0.08		
Rotamer outliers (%)	0.37		
Clashscore	3.24		
Average B-factor	48.9		
macromolecules	49.3		
ligands	46.6		
solvent	42.8		
Number of TLS groups	6		

Table S 5. Specific activity in hydrogen evolution of data presented in the main text: activity measured in hydrogen evolution in solution assay presented as μmol H_2/mg/min. Values presented are averages of three replicates, along with standard deviation.

**Enzyme**	**Anaerobic**	**After 4 hours of air exposure**	**After 24 hours of air exposure**
*Cb*A5H WT	360.7 (±82)	222 (±18.3)	145.2 (±24)
*Cb*A5H C236A	403 (±43)	180.8 (±5.3)	163 (±20.2)
*Cb*A5H H245A	405 (±19)	73.5 (±3.5)	39 (±0.5)
*Cb*A5H C236A/ H245A	1155.5 (±79.2)	164.8 (±12.5)	94.4 (±10.2)
*Ca*HydA1	730.3 (±35)	107 (±9.3)	51 (±4.7)
*Cn*HydA1	61.8 (±11.7)	41.4 (±3.6)	21.5 (±1.6)

Table S 6. Sequence identity between CnHydA1, CbA5H, and CpI.

**Enzyme**	**Sequence identity to *Cn*HydA1**		
	**H+Fd domains**	**H domain only**	**Fd domain only**
***Cb*A5H**	48.2	48.75	45
***Cp*I**	45.2	47.78	29.4

Table S 7. H_{ox} and H_{inact} FTIR profile of CbA5H, CnHydA1, DdH, CpIII, and ToHydA. Values presented are the peak maxima corresponding to the vibrational stretches of CO and CN in the H-cluster of the FeFe hydrogenases.

**Enzyme**	**Hox**	**H_{inact}**	**Hox-CO**	***Ref.***
***Cb*A5H**	2091,2080,1964,1940,1800	2107,2080,2011,1992,1840	2094,2090,2016,1971,1963,1807	[5]
***Cn*HydA1**	2086,2077,1967,1947, 1802	2108,2077,2011,1995,1847	2096,2086,2011,1982,1967,1809	(This work)
***Dd*H**	2094,2079,1965,1940,1802	2106,2086,2007,1983,1847	2096,2089,2017,1972,1963,1812	[15]
***Cp*III**	2093, 2073, 1967, 1948, 1783	2107,2083,1999,1985,1822	2103,2090,2009,1978,1959,1795	[16]
***To*HydA**	N.D.	2101,2081,1996,1975,1826	N.D.	[17]

Table S 8. The lower and upper potentials applied during each phase of the potential step experiments, following re-activation at – 0.800 V vs Ag/AgCl. Applied potential phases were randomised to prevent bias resulting from film loss over the course of the experiment. See Figure S 19 for the complete potential-time profile and raw data output.

Phase Number	1	2	3	4	5	6	7	8	9	10	11	12	13	14	15
Upper Potential (V vs Ag/AgCl)	-0.545	-0.4325	-0.4075	-0.445	-0.4825	-0.5575	-0.57	-0.4575	-0.47	-0.5325	-0.42	-0.395	-0.5075	-0.52	-0.495
Lower Potential (V vs Ag/AgCl)	-0.445	-0.3325	-0.3075	-0.345	-0.3825	-0.4575	-0.47	-0.3575	-0.37	-0.4325	-0.32	-0.295	-0.4075	-0.42	-0.395

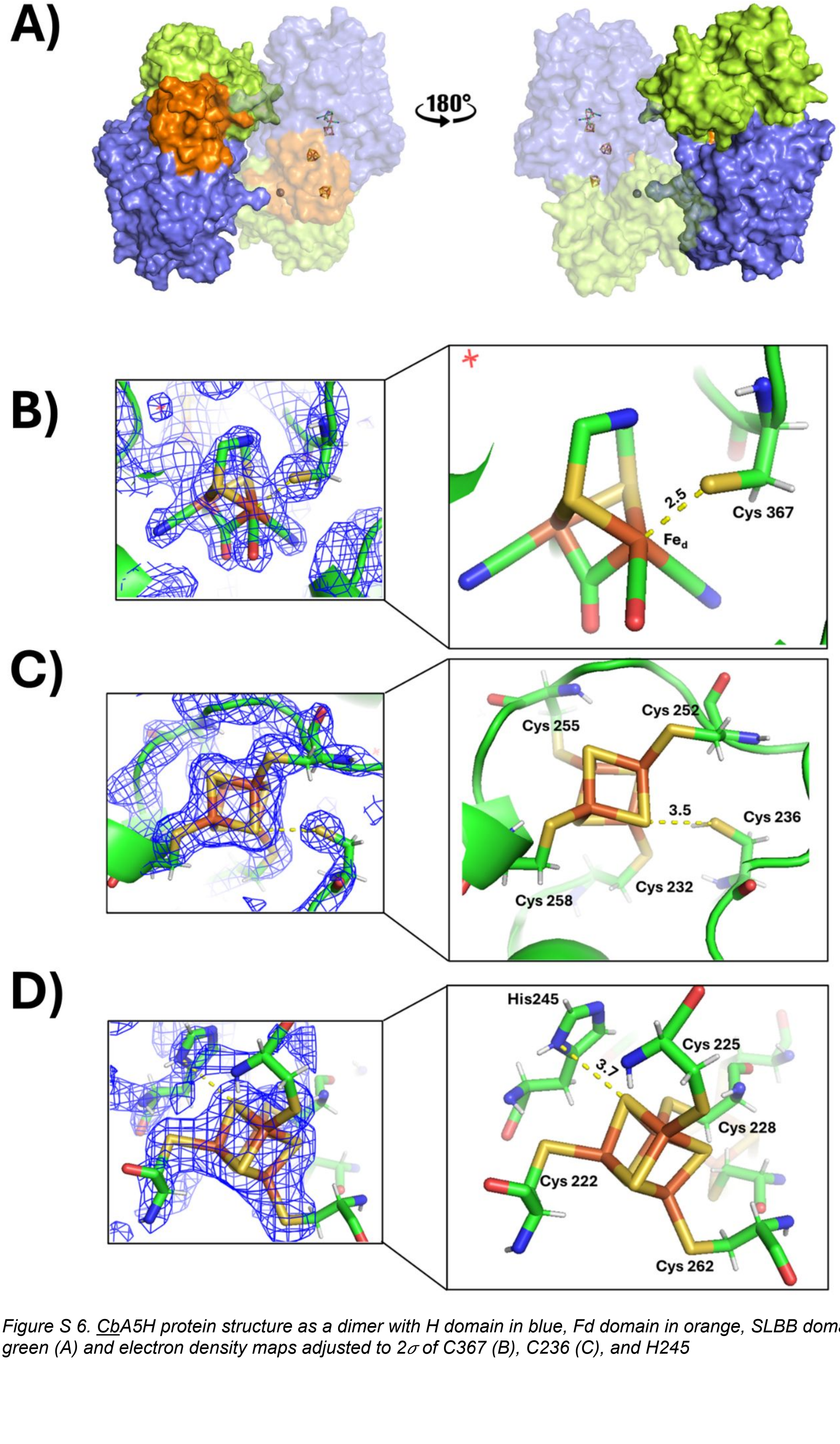

Figure S 6. CbA5H protein structure as a dimer with H domain in blue, Fd domain in orange, SLBB domain in green (A) and electron density maps adjusted to 2σ of C367 (B), C236 (C), and H245

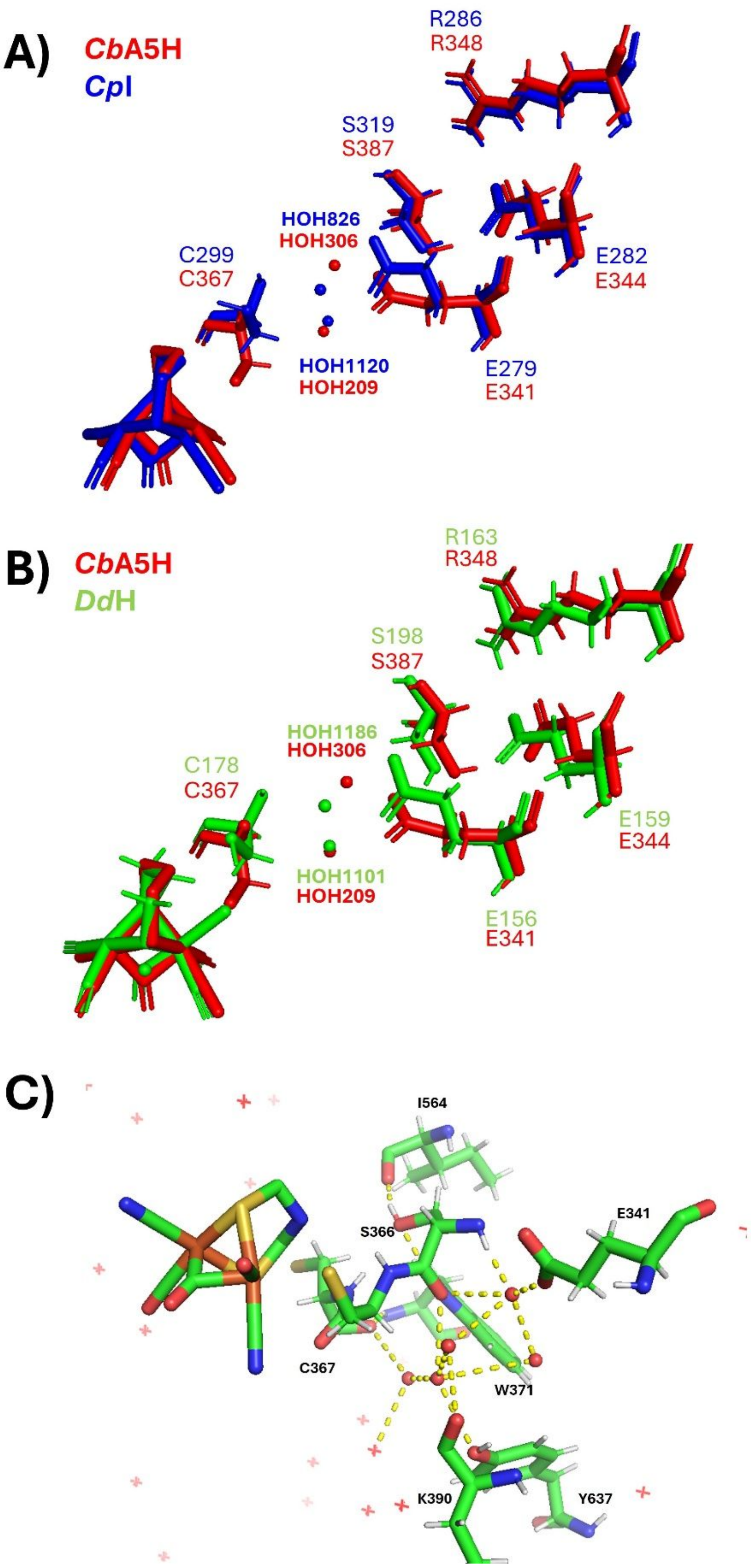

Figure S 7. The proton transfer pathway of <u>Cb</u>A5H and H bonding network required to stabilize the TSC loop. A) shows the proton transfer pathway in the aligned structures of <u>Cb</u>A5H in the H_{inact} state (water numbering is of chain A) and CpI in the H_{ox} state (PDB: 4XDC), B) shows the proton transfer pathway in the aligned structures of CbA5H in the cysteine-bound H_{inact} state (water numbering is of chain A) and DdH in the sulfide-bound H_{inact} state (PDB: 6SG2). C) shows the H bonding network required to stabilize the TSC loop in H_{inact} state in <u>Cb</u>A5H, which reveals the involvement of five water molecules in addition to I564, S366, and W371 stabilizing the loop directly, and E341, K390 and Y637 stabilizing the loop indirectly by bonding the water molecules in the network.

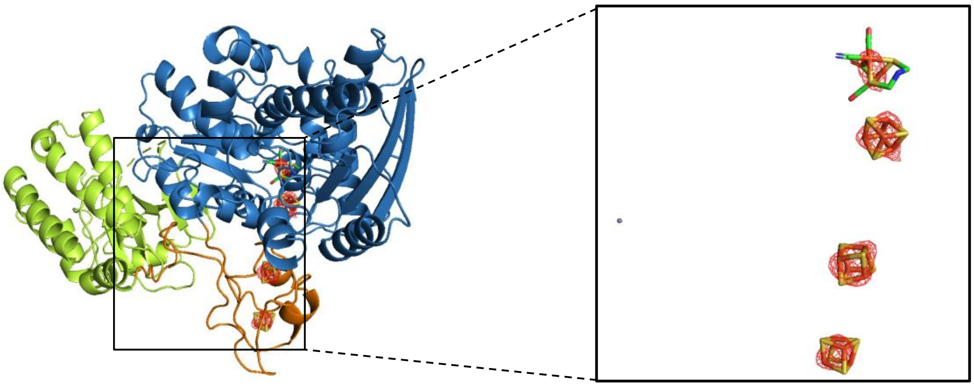

Figure S 8. The anomalous diffraction data of <u>Cb</u>A5H collected at the absorption edge of iron. The figure shows the crystal structure of <u>Cb</u>A5H monomer (colour coded: H domain (blue), Fd domain (orange), SLBB domain (green)), which is zoomed at the metal content with the diffraction map (map adjusted to 9 σ, coloured red), without the protein scaffold.

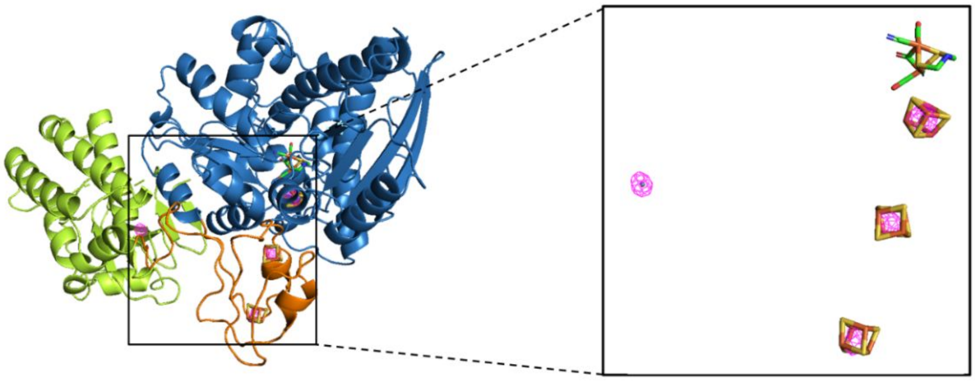

Figure S 9. The anomalous diffraction data of <u>Cb</u>A5H collected at the absorption edge of zinc. The figure shows the crystal structure of <u>Cb</u>A5H monomer (colour coded: H domain (blue), Fd domain (orange), SLBB domain (green)), which is zoomed at the metal content with the diffraction map (map adjusted to 9 σ, coloured purple), without the protein scaffold.

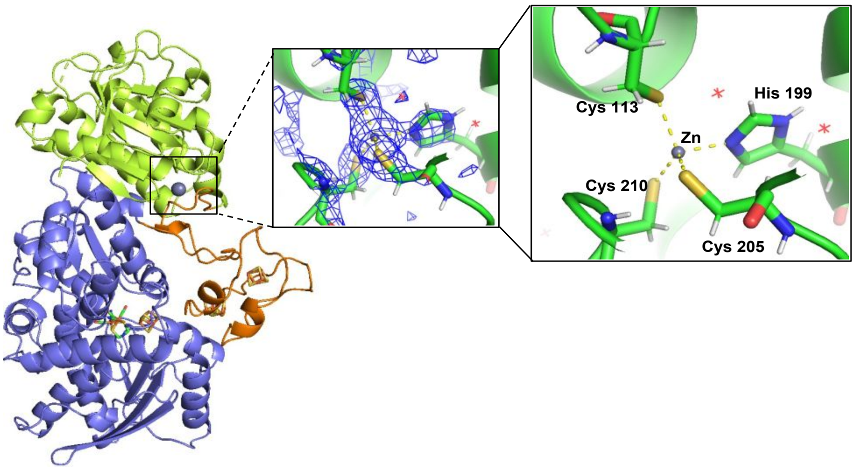

Figure S 10. The location of zinc ion in <u>Cb</u>A5H. The figure shows the crystal structure of a <u>Cb</u>A5H monomer (colour coded: H domain (blue), Fd domain (orange), SLBB domain (green)), zoomed to illustrate the density map of the coordinated residues (map adjusted to 3 σ), then zoomed once again to show the identity of the residues without the map.

```
DdH       ------------------------------------------------------------   0
CpI       ------------------------------------------------------MK-----  2
CnHydA1   ------------------------------------------------------------   0
CbA5H     MGDNKKSFIQSALGSVFSVFSEDELKELSNGRKIAICGKVNNPGIIEVPEGATLNEIIQL   60

DdH       ------------------------------------------------------------   0
CpI       -TIIINGVQFNTDED-TTILKFARDNNIDI--SALCFLNNCNNDI--NKCEICTVEVEGT   56
CnHydA1   ------------------------------------------------------------   0
CbA5H     CGGLINKSNFKAAQIGLPFGGFLTEDSLDKEFDFGLFYENIARTIIVLSQEDCIIQFEKF   120

DdH       ------------------------------------------------------------   0
CpI       GLVTACDTLIEDGMIINTNSDAVNEKIKSRISQLLDIH-EFKCGPCNRRENCEFLKLVIK   115
CnHydA1   ------------------------------------------------------------   0
CbA5H     YIE-YLLAKIKDGSYKN------YEVVKEDITEMFNILNRISKGVSNMREIYLLRNLAVT   173

DdH       ------MSRTVMERIEYEM------HTPDPK----ADPDKLHFVQIDEAKCIGCDTCSQY   44
CpI       YKAR--ASKPFLPKDKTEYVDE-----------------RSKSLTVDRTKCLLCGRCVNA   156
CnHydA1   --------------MI----DSSNKNKTNTSCTESNTDLNDIFYFQITEKCVGCTKCARV   42
CbA5H     VKSKMNQKHNIMEEIIDKFYEEIEEHIEEKKCY--TSQCNHLVKLTITKKCIGCGACKRA   231
                                                  .         **: *  * .

DdH       CPTAAIFGEM-------------GEPHSIPHIEACINCGQCLTHCPENAIYEAQSWVPEV   91
CpI       CGKNTETYAMKFLNKNGKTIIGAEDEK-CFDDTNCLLCGQCIIACPVAALSEK-SHMDRV   214
CnHydA1   CPVSCISGK-------------VKERH-VIDTTKCVKCGQCISACPMGALPKI-NFISEA   87
CbA5H     CPVDCIDGE-------------LKKKH-EIDYNRCTHCGACVSACPVDAISAG-DNTMLF   276
          *                    . :       *  ** *:  **  *:     .
              C236                H245

DdH       EKKLKDGKVKCIAMPAPAVRYALGDAFGMPVGSVTTGKMLAALQKLGFAHCWDTEFTADV   151
CpI       KNALNAPEKHVIVAMAPSVRASIGELFNMGFGVDVTGKIYTALRQLGFDKIFDINFGADM   274
CnHydA1   KKALNQKDKLVITQVAPAIRATLGEYFGLEPGTLVTGKMVAALRAIGFDKVFDTDFAADL   147
CbA5H     LRDLATPNKVVITQMAPAVRVAIGEAFGFEPGENVEKKIAAGLRKLGVDYVFDTSWGADL   336
           . *   .   *.  **::* ::*: *.:  *  .  *: :.*: :*.   :* .: **:

DdH       TIWEEGSEFVERLTK----KSDMPLPQFTSCCPGWQKYAETYYPELLPHFSTCKSPIGMN   207
CpI       TIMEEATELVQRIEN------NGPFPMFTSCCPGWVRQAENYYPELLNNLSSAKSPQQIF   328
CnHydA1   TIMEEATEFAHRLKH------GGKLPILTSCCPGWINFFEHDFRDLADIPSSCKSPQQMF   201
CbA5H     TIMEEAAELQERLERHLAGDESVKLPILTSCCPSWIKFIEQNYGDMLDVPSSAKSPMEMF   396
          ** **.:*: .*: .         .  :* :***** *      *  : ::   *:.***  :
                                      L364         M382       P386

DdH       GALAKTYGAERMKYDPKQVYTVSIMPCIAKKYEGLRPELKSSGMRDIDATLTTRELAYMI   267
CpI       GTASKTYYPSISGLDPKNVFTVTVMPCTSKKFEADRPQMEKDGLRDIDAVITTRELAKMI   388
CnHydA1   GTIAKTYFAQKMNIEPKDIIVVSIMPCLAKKYEAARPEMTTDGTRDVDIVISTQELAELI   261
CbA5H     AIVAKEIWAKEKGLSRDEVTSVAIMPCIAKKYEASRAEFSVEMNYDVDYVITTRELIKIF   456
          .   :*     .     . .::  *::*** :**:*. * ::  .   *:* .::*:**  ::

DdH       KKAGIDFAKLPDGKRDSLMGESTGGATIFGVTGGVMEAALRFAYEAVTGKKPDSWDFKAV   327
CpI       KDAKIPFAKLEDSEADPAMGEYSGAGAIFGATGGVMEAALRSAKDFAENAELEDIEYKQV   448
CnHydA1   KENNIDFNSLEDDDFDKPLGESSGAGTIFGTSGGVAEAAIRTAYELITNETIPALEFKGV   321
CbA5H     ENSGINLKEIEDEEIDTVMGEYTGAGIIFGRTGGVIEAATRTALEKMTGERFDNIEFEGL   516
          :.  * : .: * . *  :** :*.. *** :*** *** * * :    .      ::: :

DdH       RGLDGIKEATVNVGGTDVKVAVVHGAKRFKQVCDDVKAGKSPYHFIEYMACPGGCVCGGG   387
CpI       RGLNGIKEAEVEINNNKYNVAVINGASNLFKFMKSGMINEKQYHFIEVMACHGGCVNGGG   508
CnHydA1   RGLNGLKEAEVEINGEKIKIAIANGLATARELLNSIRNGEKNYHLIEIMACQGGCIGSGM   381
CbA5H     RGWDGFRVCELEAGDIKLRIGVAHGLREAAKMLDKIRSGEEFFHAIEIMACVGGCIGGGG   576
          ** :*:: . :: .. . .:.: :*     :. ..    .:. :* ** *** ***: .*
                                                       A561

DdH       QPVMPGVL----EAMDRTTTRLYAGLKKRLAMAS-ANKA--------------------   421
CpI       QPHVNPKDLEKVDIKKVRASVLYNQDEHLSKRKSHENTALVKMYQNYFGKPGEGRAHEIL   568
CnHydA1   QPFIKNH----CNIIQKRSEALYSEDRHKTLRKSHENPYVLKIYEEFLGEPYGEKAHKYL   437
CbA5H     QPKTKGNK---QAALQKRAEGLNNIDRSKTLRRSNENPEVLSIYEKYLDHPLSNKAHELL   633
          **                .  :  *     .        *  *

DdH       -----------      421
CpI       HFKYKK-----      574
CnHydA1   HTEYVK-----      443
CbA5H     HTVYFPRVKKD      644
```

Figure S 11. Sequence alignment of CbA5H with DdH, CpI, and CnHydA1 where the red highlight is the conserved motifs of FeFe hydrogenases [18], the yellow highlight are the residues suggested to confer the ability to form H_{inact} redox state [12], the blue highlight shows M382 in CbA5H, the purple highlight C236 and H245 mentioned in main text. Boxes show the residue numbers in CbA5H, while the green highlights the H352, L353,

A354, G355, D356, and E357 an insertion in Cb̲A5H that is absent in oxygen sensitive FeFe hydrogenases as well Cn̲HydA1.

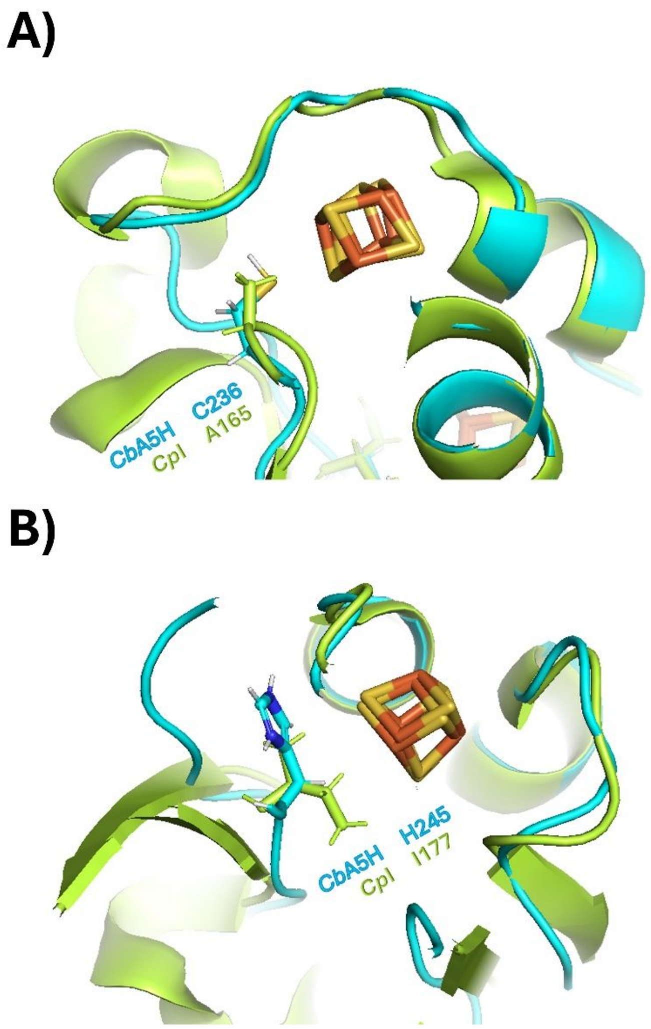

Figure S 12. Structural alignment of the Fd domains of CbA5H (blue) and CpI (green) (PDB:6N59) presenting the corresponding residue to C236 in CbA5H in the proximal cluster to be A165 in CpI (A) and corresponding residue to H245 in the distal cluster to be I177 in CpI (B).

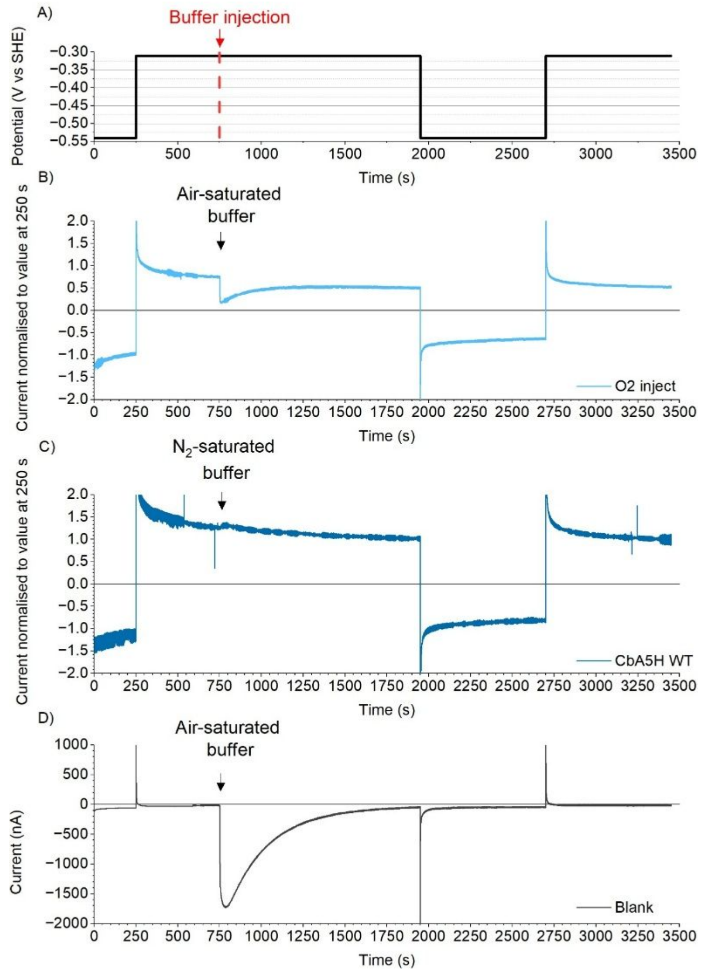

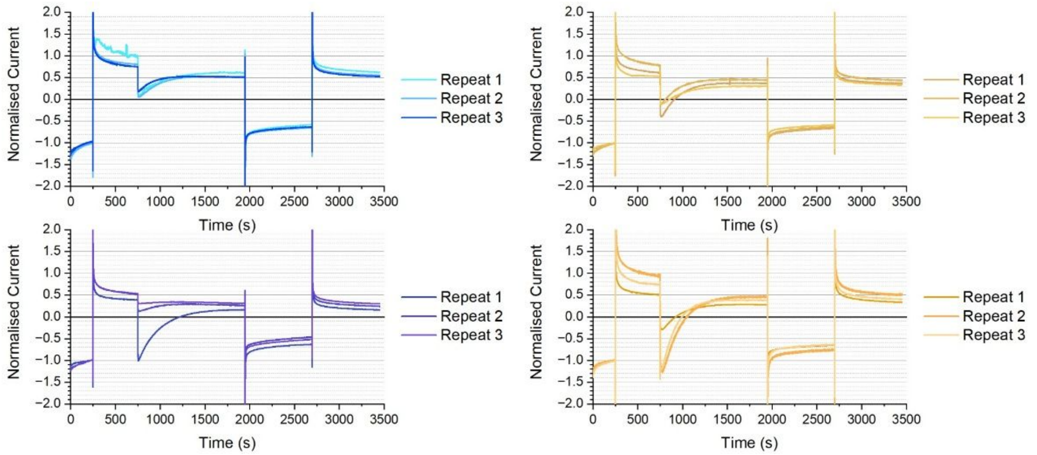

	% recovered activity			Avg %	Std error
	Run 1	Run 2	Run 3		
WT, O_2	60	69	73	67	4
C236A, O_2	56	60	62	59	2
H245A, O_2	55	48	38	47	5
C236A/H245A, O_2	57	53	62	57	2
WT, N_2	77				

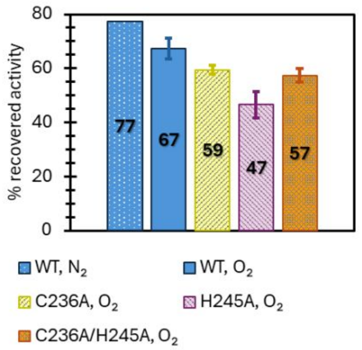

Figure S 13. Gas-saturated buffer injection electrochemistry experiments conducted at pH 8.0, 5 °C. (Left panel) (A) potential-step profile applied to the working electrode; "buffer injection" indicates that at 750 s a 1.47 ml injection of gas-saturated buffer is made into the electrochemical cell solution of 6.69 ml H_2-saturated buffer, with an active flow of 100% H_2 and electrode rotation rate of 1, 000 rpm. (B) & (C) Normalised-current from air- & N_2-saturated Cb A5H WT experiment, as indicated. Current is normalised to the level recorded at 250 s. (D) Enzyme-free ("blank") air-injection experiment to visualised gas-purging. (Top-right panel) Normalised current response to injection of air-saturated buffer of three different electrode-enzyme films of Cb A5H WT (blue, top left); C236A (yellow, top right); H245A (purple, bottom left) and C236A/H245A (orange, bottom right). (Bottom-right panel) H_2-oxidation current recovered at the end of experiments (3250 – 3450 s) as a percentage of the activity measured just prior to gas-saturated buffer injection (650 - 750 s).

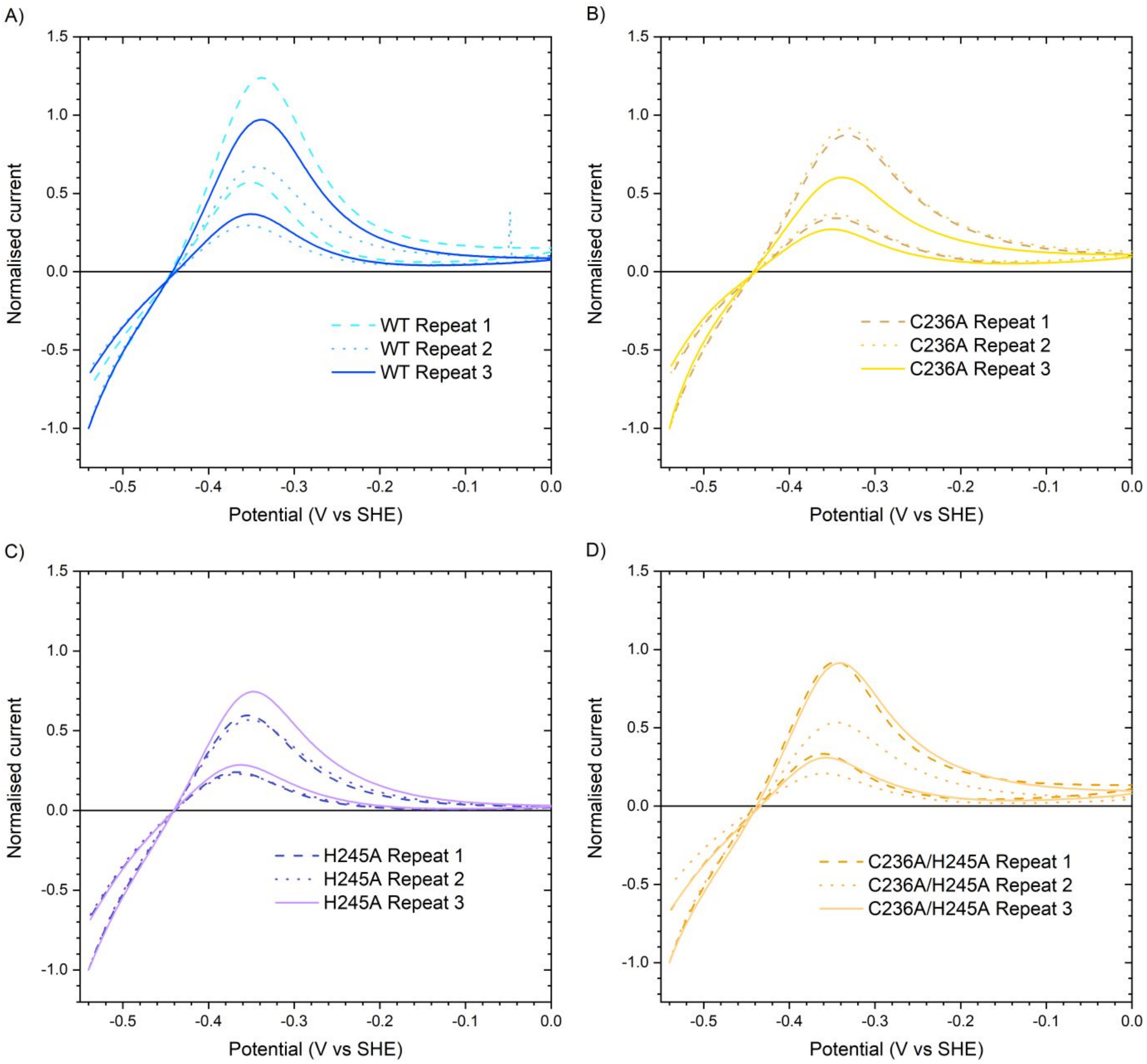

Figure S 14. Scan 1 of repeat pH 8.0, 100% H_2 atmosphere Cb A5H 3 mV s^{-1} cyclic voltammetry measurements with the current normalised to the starting value. (A) WT (blue); (B) C236A (yellow); (C) H245A (purple); (D) C236A/H245A (orange). The first repeat is represented by a dashed line, the second by a dotted line and the third by a solid line. Other experimental conditions: 5 °C; electrode rotation rate 3,000 rpm.

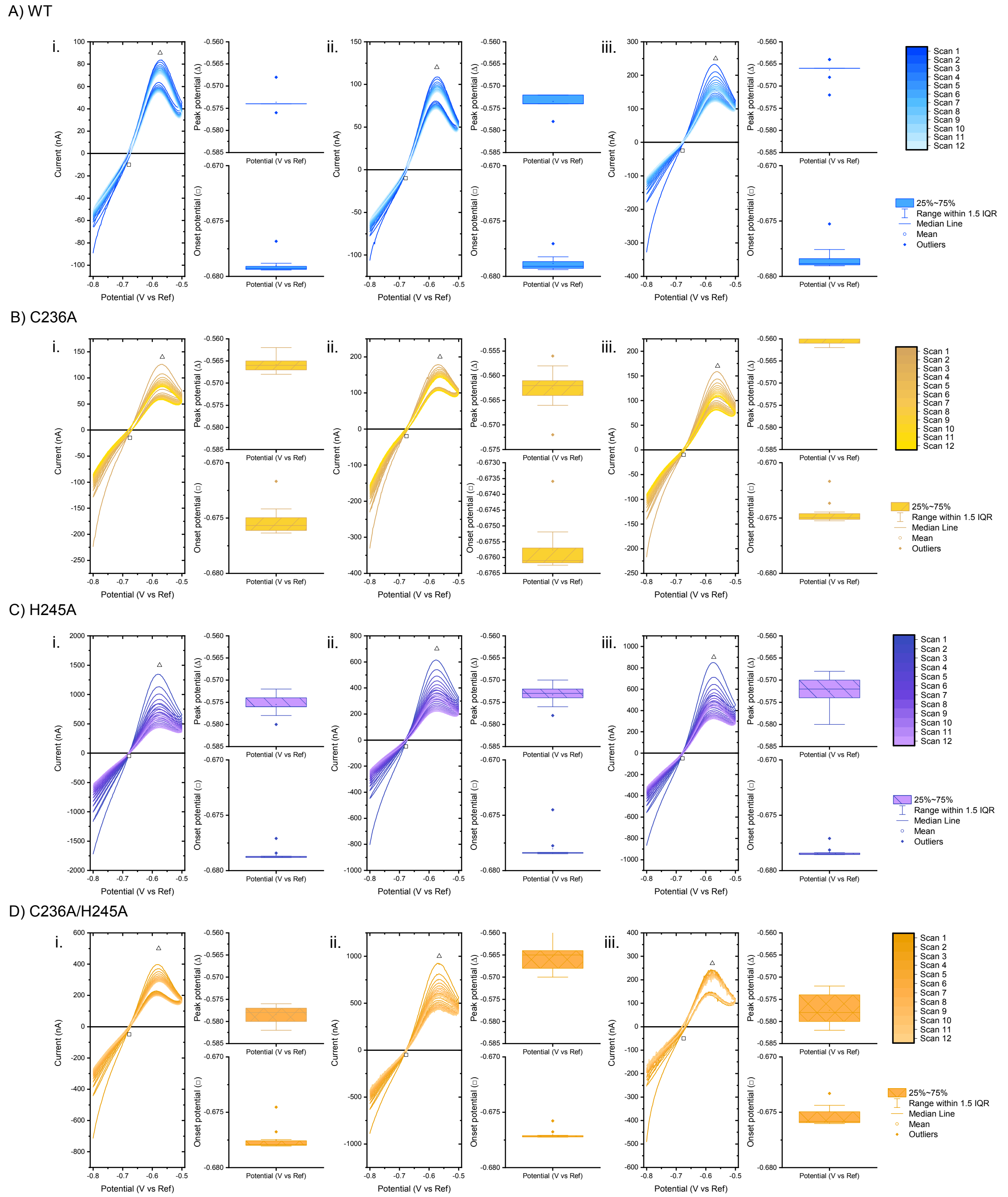

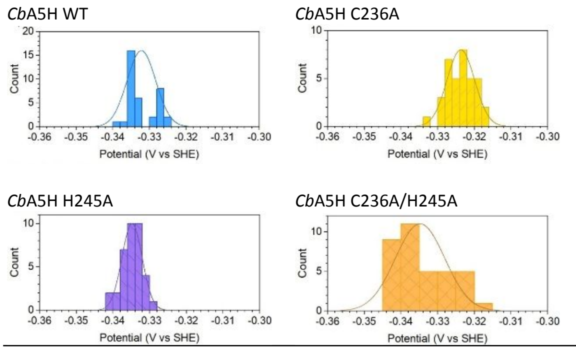

Figure S 15. (Top panel) CbA5H consecutive 3 mV s^{-1} cyclic voltammograms scanning from -0.8 to -0.5 V vs Ref (Ref = Ag/AgCl) at pH 8.0 and under a 100% H_2 atmosphere for (i to iii) different electrode-enzyme films of (A) WT (blue); (B) C236A (yellow); (C) H245A (purple); (D) C236A/H245A (orange). The intensity of the colour depicts the relative scan number, as indicated. The box plots summarise the scan-to-scan variability in zero-current ("onset") and peak potential, □ and Δ, respectively. IQR = Interquartile range. (Bottom panel) Histograms of peak potentials in V vs SHE. Other experimental conditions: 5 °C; electrode rotation rate 3,000 rpm.

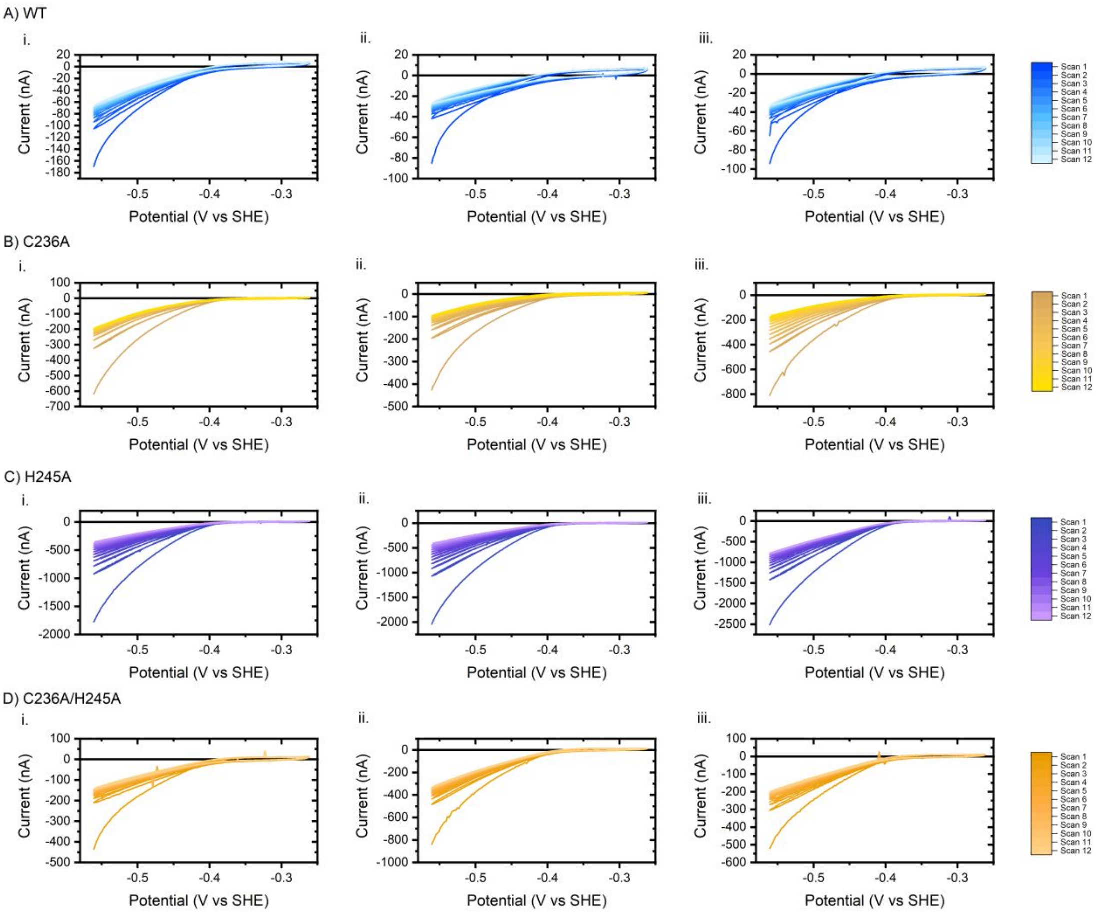

*Figure S 16. Cb*A5H *consecutive 3 mV s^{-1} cyclic voltammograms, measured at pH 8.0 and under a 100% N_2 atmosphere for (A) WT (blue); (B) C236A (yellow); (C) H245A (purple); (D) C236A/H245A (orange). The intensity of the colour depicts the relative scan number, as indicated. (i to iii) Repeat experiments on different electrode-enzyme films. Other experimental conditions: 5 °C; electrode rotation rate 3,000 rpm.*

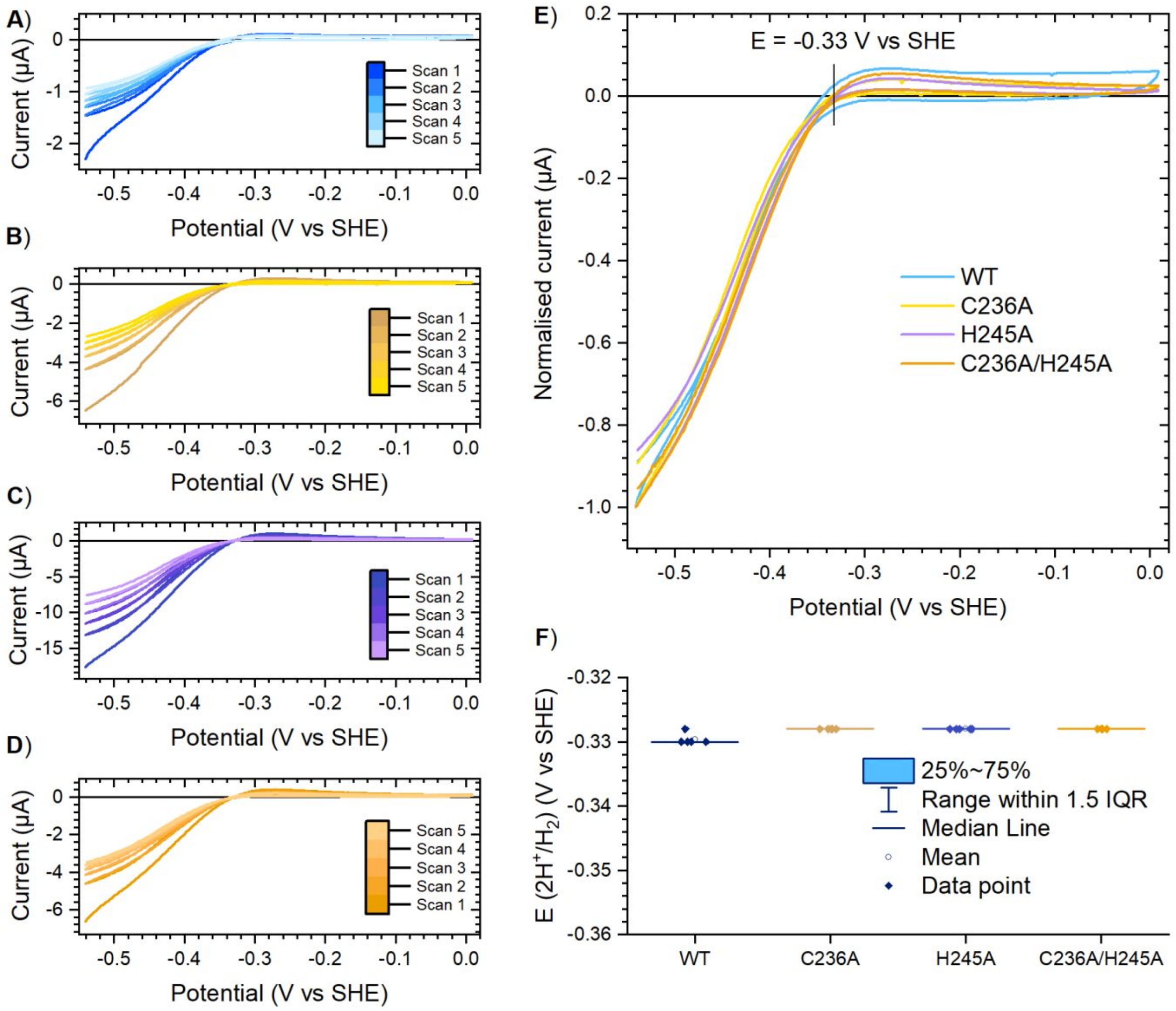

Figure S 17. <u>Cb</u>A5H consecutive 3 mV s^{-1} cyclic voltammograms measured at pH 6.0 under a 100% H_2 atmosphere for (A) WT (blue); (B) C236A (yellow); (C) H245A (purple); (D) C236A/H245A (orange). The intensity of the colour depicts the relative scan number, as indicated. (E) Scan 5 for all variants plotted on the same graph, with current normalised to the value measured at the start of the experiment. (F) Box plots summarise the scan-to-scan variability in the E($2H^+/H_2$) zero-current potential values extracted from data in A)-D). IQR = Interquartile range. Other experimental conditions: 5 C; electrode rotation rate: 3, 000 rpm.

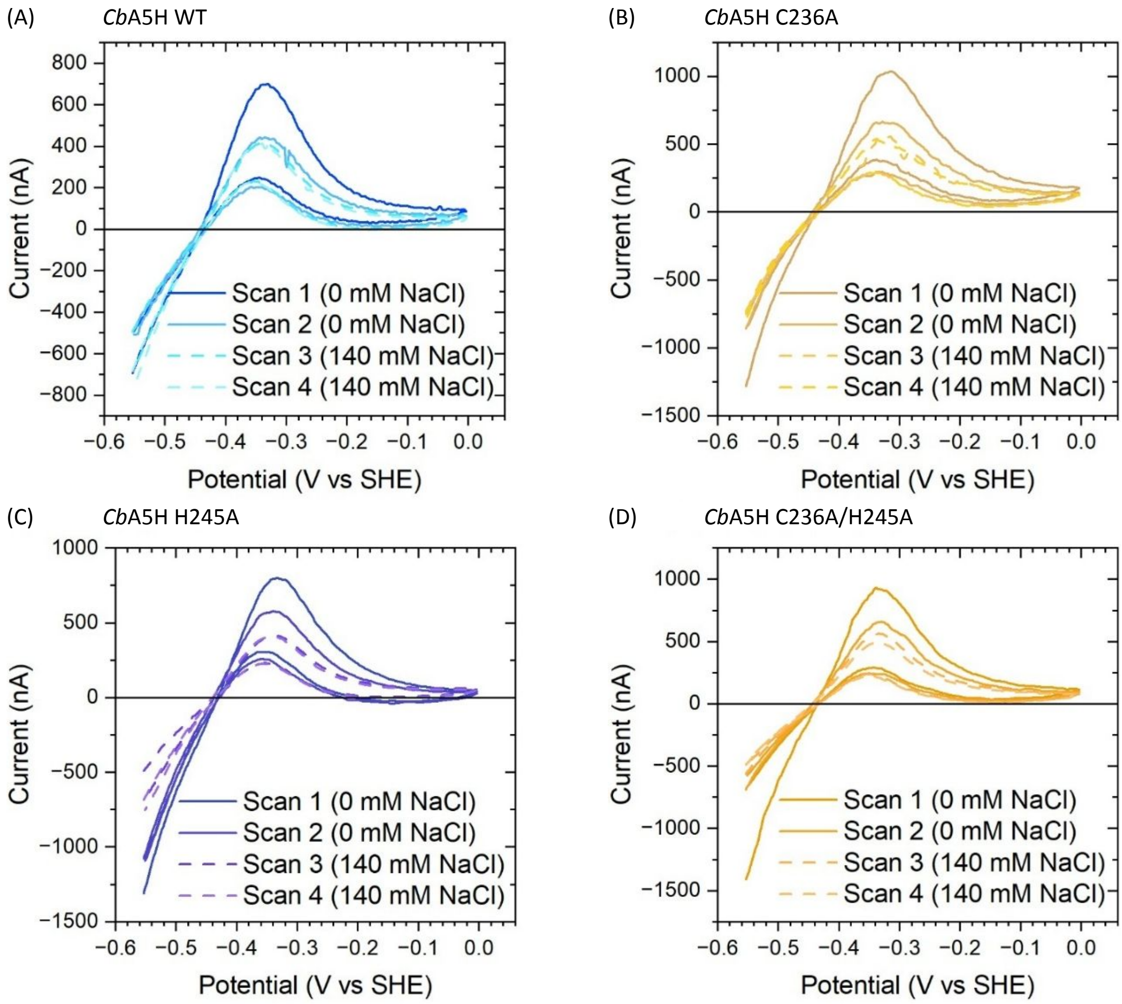

Figure S 18. Cb<u>Cb</u>A5H continuous 3 mV s^{-1} cyclic voltammetry experiments scanning from -0.55 to 0 V vs SHE measured in the absence (scans 1 and 2) and then presence (scans 3 and 4) of NaCl at pH 8.0 under a 100% H_2 atmosphere for (A) WT (blue); (B) C236A (yellow); (C) H245A (purple); (D) C236A/H245A (orange). In all scans the buffer contained 140 mM Na_2SO_4 and 5 mM of each of HEPES, sodium acetate, TAPS, CHES and MES; in scans 3 and 4 extra buffer containing 1 M NaCl was added to the cell solution, sufficient to give a final concentration of 140 mM NaCl. Other experimental conditions: 5 C; electrode rotation rate: 3, 000 rpm.

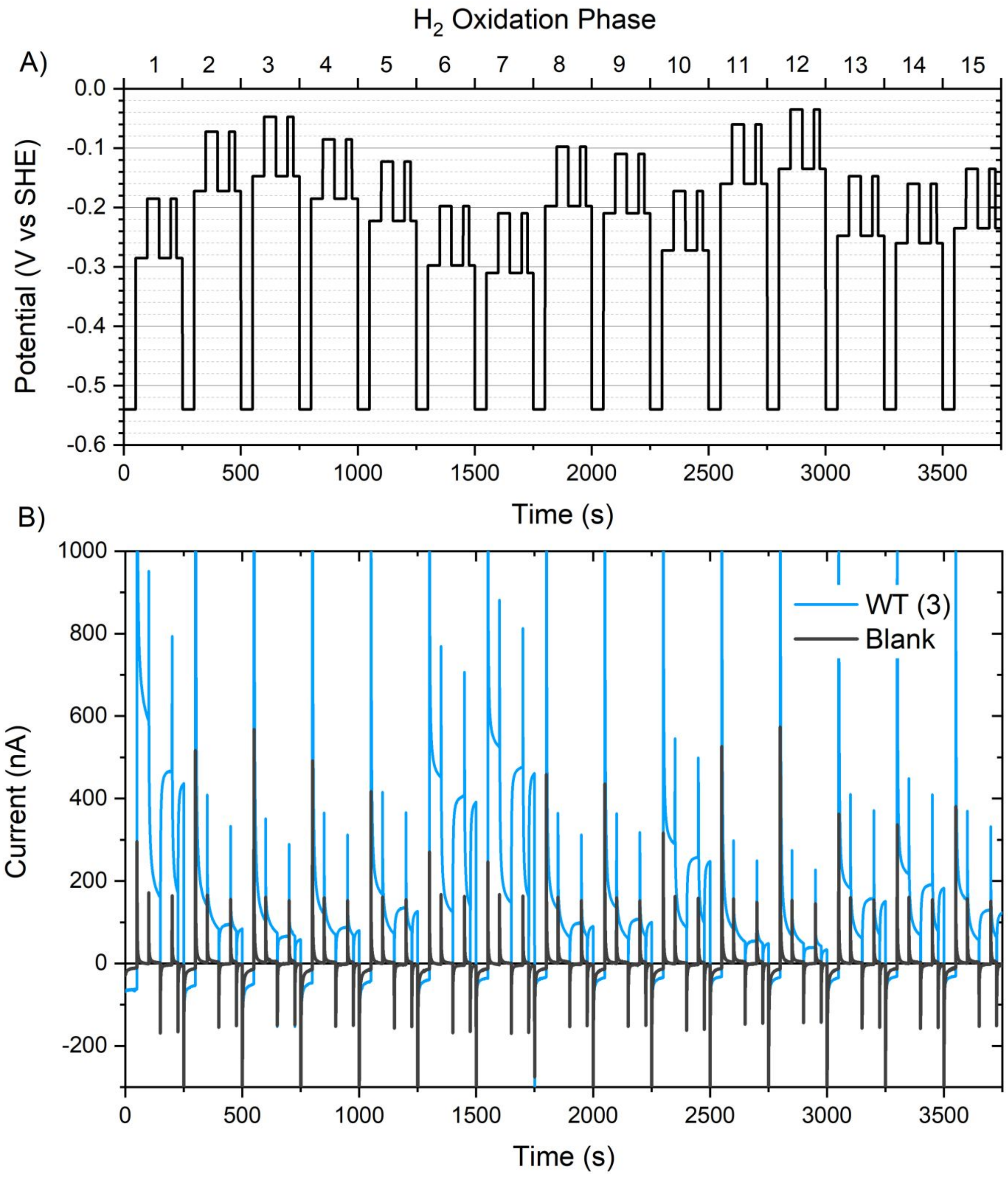

Figure S 19. A) Potential-time sequence used to probe CbA5H anaerobic inactivation and B) corresponding current-time response for both a WT (blue) and enzyme-free ("blank", grey) experiment. The "WT (3)" label denotes that this was the third repeat, see Figure S 20. Other experimental conditions: pH 8.0; 100 % H_2 atmosphere; 5 °C; electrode rotation rate 3,000 rpm.

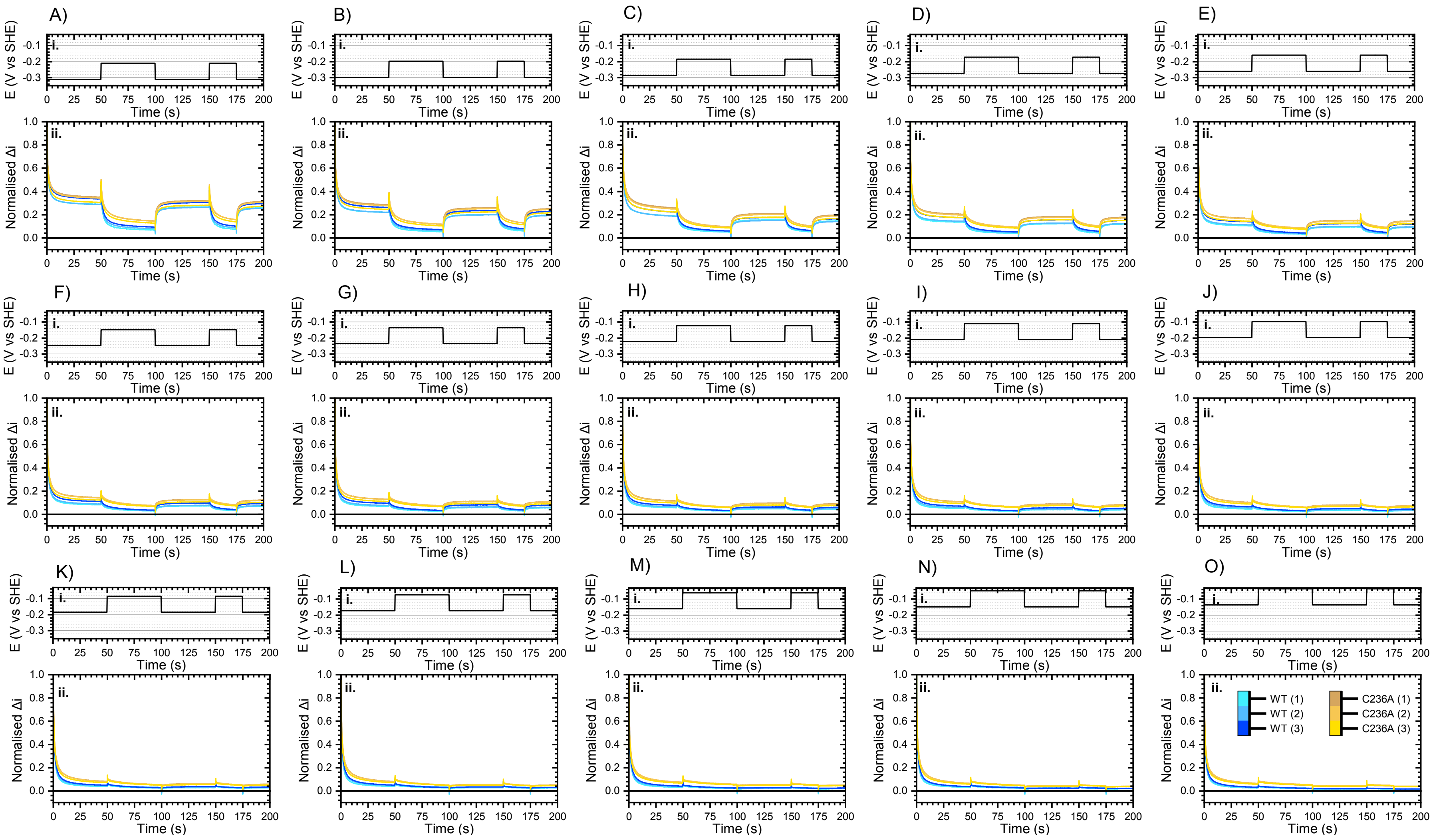

Figure S 20. Summary of <u>*Cb*</u>*A5H WT anaerobic inactivation experiments conducted at pH 8.0 and under 100 % H_2 atmosphere. The total experimental data has been separated into the individual H_2 oxidation phases, as shown in A)-O). The panels are ordered by increasing inactivation/ reactivation potential and within each panel (i.) the relevant potential-time and (ii.) blank-corrected, normalised current ("Normalised Δi") is shown with t = 0 as the start time of that phase of the experiment. Data is from three different electrode-enzyme films of WT (blue) and C236A (yellow). Other experimental conditions: 5 °C, electrode rotation rate: 3,000 rpm.*

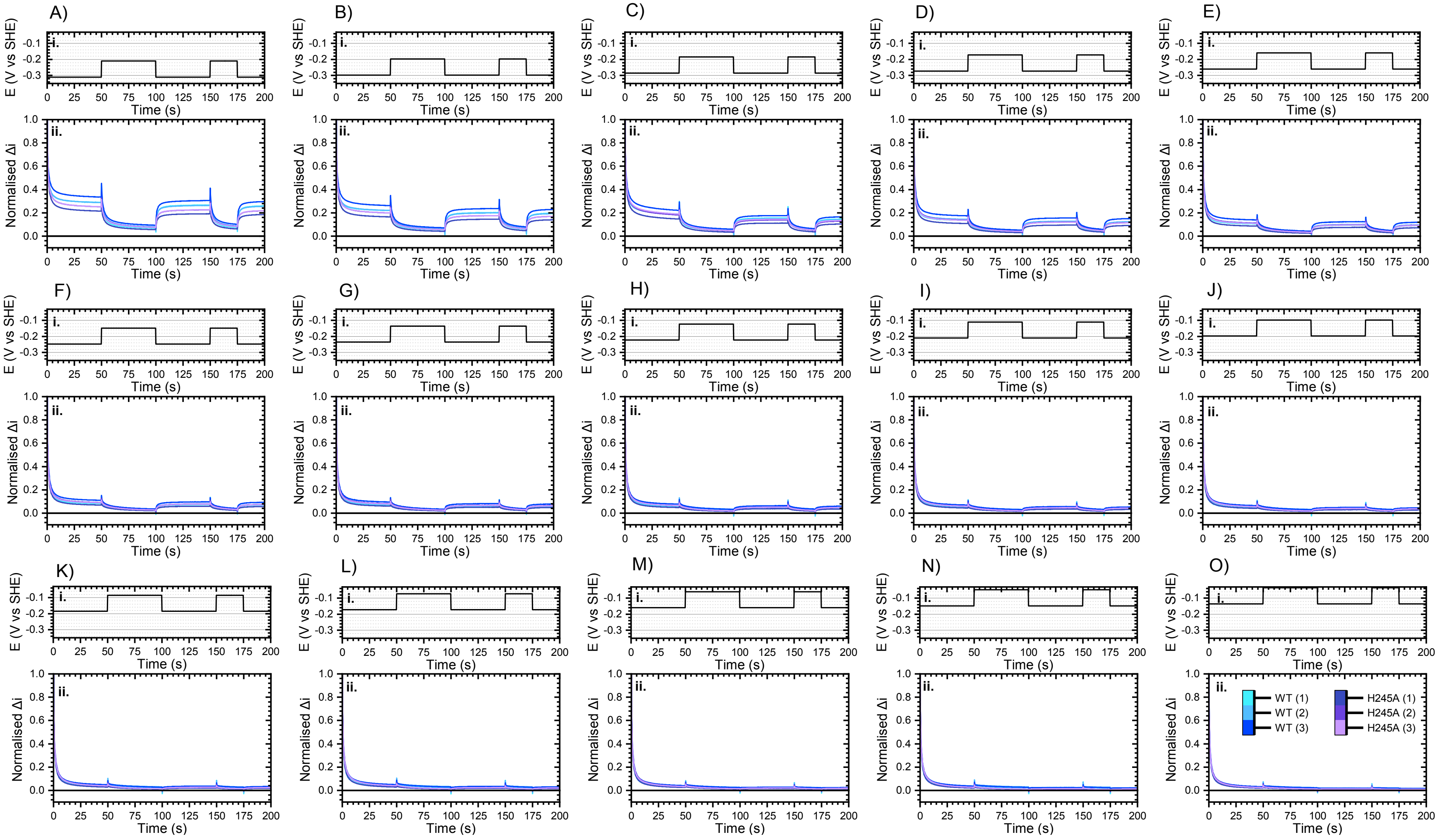

Figure S 21. Summary of CbA5H WT anaerobic inactivation experiments conducted at pH 8.0 and under 100 % H_2 atmosphere. The total experimental data has been separated into the individual H_2 oxidation phases, as shown in A)-O). The panels are ordered by increasing inactivation/ reactivation potential and within each panel (i.) the relevant potential-time and (ii.) blank-corrected, normalised current ("Normalised Δi") is shown with t = 0 as the start time of that phase of the experiment. Data is from three different electrode-enzyme films of WT (blue) and H245A (purple). Other experimental conditions: 5 °C, electrode rotation rate: 3,000 rpm.

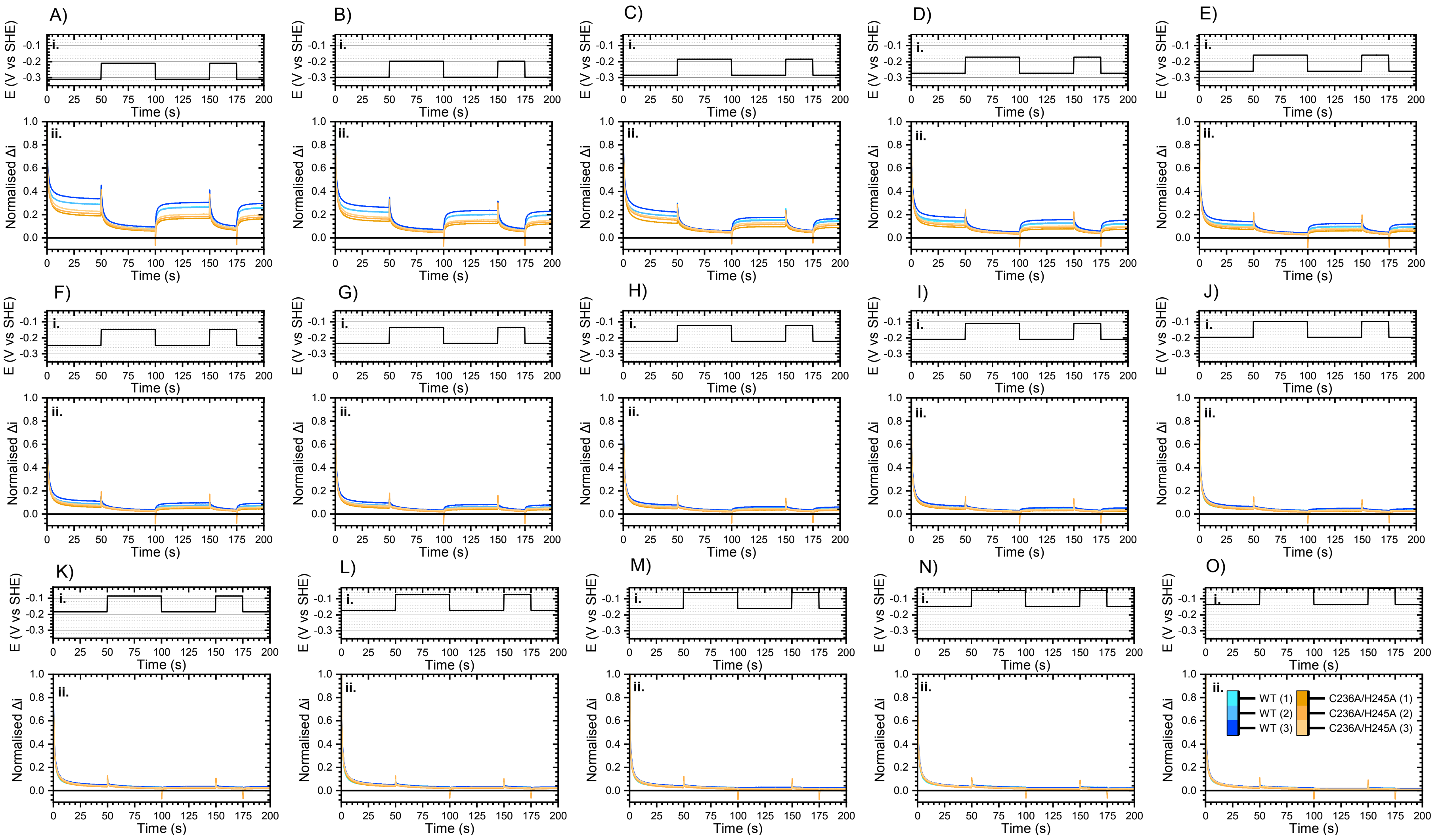

Figure S 22. Summary of <u>Cb</u>A5H WT anaerobic inactivation experiments conducted at pH 8.0 and under 100 % H_2 atmosphere. The total experimental data has been separated into the individual H_2 oxidation phases, as shown in A)-O). The panels are ordered by increasing inactivation/ reactivation potential and within each panel (i.) the relevant potential-time and (ii.) blank-corrected, normalised current ("Normalised Δi") is shown with t = 0 as the start time of that phase of the experiment. Data is from three different electrode-enzyme films of WT (blue) and C236A/H245A (orange). Other experimental conditions: 5 °C, electrode rotation rate: 3,000 rpm.

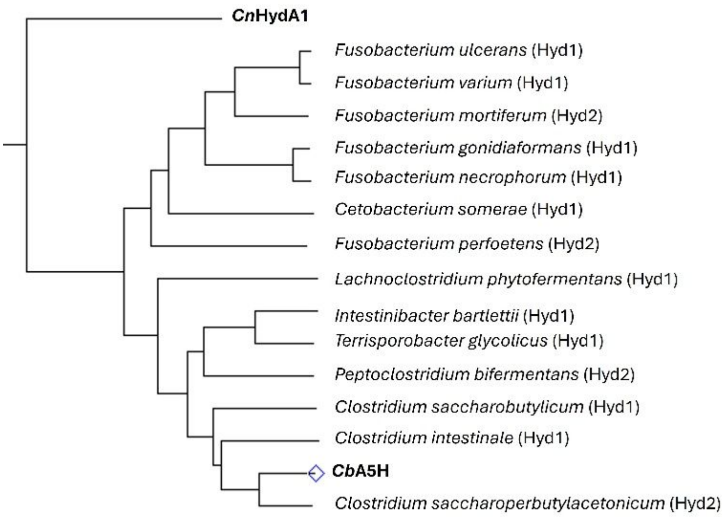

Figure S 23. The phylogenetic distance between Cn HydA1 and Cb A5H from the phylogenetic tree by Greening et al.[18].

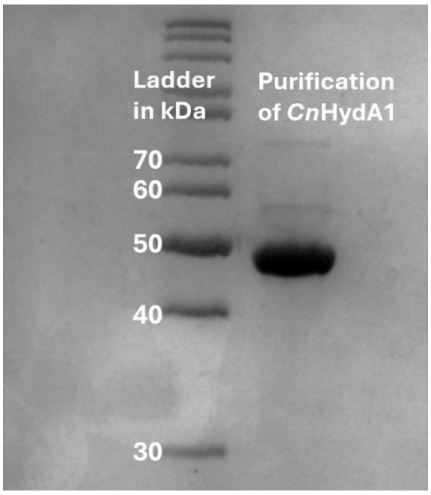

Figure S 24. SDS-PAGE analysis of Cn HydA1 purification. Protein expected size is 51.4 kDa.

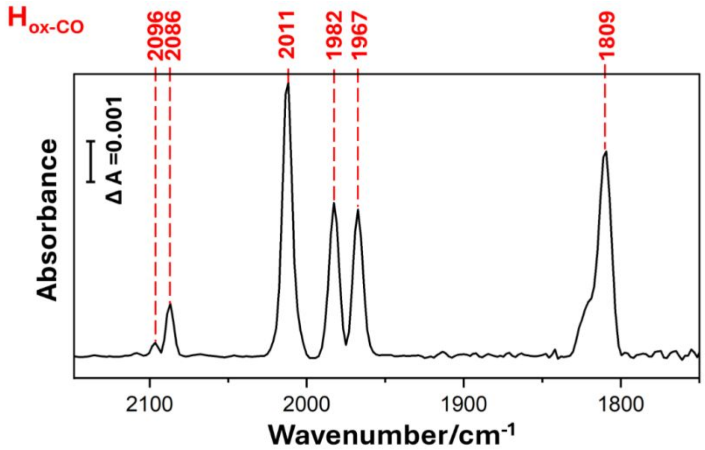

Figure S 25. The H_{ox-CO} FTIR spectrum of CnHydA1 in the range of 2150-1750 cm^{-1}. The anaerobically purified protein was sparged with 100% CO gas for 5 minutes then incubated on ice for an hour. This measurement was obtained using Bruker Invenio R FTIR equipped with DLaTGS detector, at spectral resolution of 4 cm^{-1}, and 512 scans.

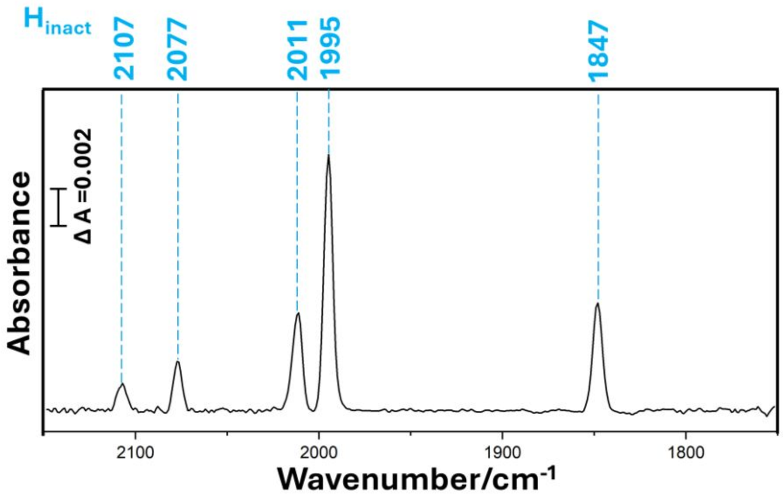

Figure S 26. The FTIR spectrum of aerobically purified CnHydA1 in the range of 2150-1750 cm^{-1}.

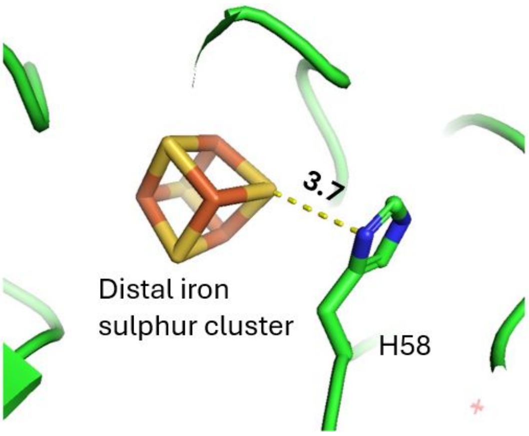

Figure S 27. The distance between H58 and the distal iron sulphur cluster in DdH (PDB:6SG2)

Supplementary references

1) Madeira, F.; Madhusoodanan, N.; Lee, J.; Eusebi, A.; Niewielska, A.; Tivey, A. R. N.; Lopez, R.; Butcher, S. The EMBL-EBI Job Dispatcher sequence analysis tools framework in 2024. *Nucleic Acids Research* **2024**, *52* (W1), W521-W525. DOI: 10.1093/nar/gkae241 (acccessed 4/12/2025).

(2) Tamura, K.; Stecher, G.; Kumar, S. MEGA11: Molecular Evolutionary Genetics Analysis Version 11. *Molecular Biology and Evolution* **2021**, *38* (7), 3022-3027. DOI: 10.1093/molbev/msab120 (acccessed 4/12/2025).

(3) Gibson, D. G.; Young, L.; Chuang, R.-Y.; Venter, J. C.; Hutchison, C. A.; Smith, H. O. Enzymatic assembly of DNA molecules up to several hundred kilobases. *Nature Methods* **2009**, *6* (5), 343-345. DOI: 10.1038/nmeth.1318.

(4) Akhtar, M. K.; Jones, P. R. Deletion of iscR stimulates recombinant clostridial Fe-Fe hydrogenase activity and H2-accumulation in Escherichia coli BL21(DE3). *Appl Microbiol Biotechnol* **2008**, *78* (5), 853-862. DOI: 10.1007/s00253-008-1377-6 From NLM.

(5) Morra, S.; Arizzi, M.; Valetti, F.; Gilardi, G. Oxygen Stability in the New [FeFe]-Hydrogenase from Clostridium beijerinckii SM10 (CbA5H). *Biochemistry* **2016**, *55* (42), 5897-5900. DOI: 10.1021/acs.biochem.6b00780.

(6) Morra, S.; Cordara, A.; Gilardi, G.; Valetti, F. Atypical effect of temperature tuning on the insertion of the catalytic iron-sulfur center in a recombinant [FeFe]-hydrogenase. *Protein Sci* **2015**, *24* (12), 2090-2094. DOI: 10.1002/pro.2805 From NLM.

(7) Tropea, J. E.; Cherry, S.; Waugh, D. S. Expression and Purification of Soluble His6-Tagged TEV Protease. In *High Throughput Protein Expression and Purification: Methods and Protocols*, Doyle, S. A. Ed.; Humana Press, 2009; pp 297-307.
(8) Winter, G. XIA2: an expert system for macromolecular crystallography data reduction. *Journal of Applied Crystallography - J APPL CRYST* **2010**, *43*, 186-190. DOI: 10.1107/S0021889809045701/ea5113sup1.pdf.
(9) Winter, G.; Waterman, D. G.; Parkhurst, J. M.; Brewster, A. S.; Gildea, R. J.; Gerstel, M.; Fuentes-Montero, L.; Vollmar, M.; Michels-Clark, T.; Young, I. D.; et al. DIALS: implementation and evaluation of a new integration package. *Acta Crystallogr D Struct Biol* **2018**, *74* (Pt 2), 85-97. DOI: 10.1107/s2059798317017235 From NLM.
(10) McCoy, A. J.; Grosse-Kunstleve, R. W.; Adams, P. D.; Winn, M. D.; Storoni, L. C.; Read, R. J. Phaser crystallographic software. *J Appl Crystallogr* **2007**, *40* (Pt 4), 658-674. DOI: 10.1107/s0021889807021206 From NLM.
(11) Casañal, A.; Lohkamp, B.; Emsley, P. Current developments in Coot for macromolecular model building of Electron Cryo-microscopy and Crystallographic Data. *Protein Sci* **2020**, *29* (4), 1069-1078. DOI: 10.1002/pro.3791 From NLM.
(12) Winkler, M.; Duan, J.; Rutz, A.; Felbek, C.; Scholtysek, L.; Lampret, O.; Jaenecke, J.; Apfel, U.-P.; Gilardi, G.; Valetti, F.; et al. A safety cap protects hydrogenase from oxygen attack. *Nature Communications* **2021**, *12* (1), 756. DOI: 10.1038/s41467-020-20861-2.
(13) Gelman, A.; Rubin, D. B. Inference from Iterative Simulation Using Multiple Sequences. *Statistical Science* **1992**, *7* (4), 457-472, 416.
(14) Cleary, S. E.; Hall, S. J.; Galan-Bataller, R.; Lurshay, T. C.; Hancox, C.; Williamson, J. J.; Heap, J. T.; Reeve, H. A.; Morra, S. Scalable Bioreactor Production of an O2-Protected [FeFe]-Hydrogenase Enables Simple Aerobic Handling for Clean Chemical Synthesis. *ChemCatChem* **2024**, *16* (16), e202400193. DOI: https://doi.org/10.1002/cctc.202400193.
(15) Rodríguez-Maciá, P.; Reijerse, E. J.; van Gastel, M.; DeBeer, S.; Lubitz, W.; Rüdiger, O.; Birrell, J. A. Sulfide Protects [FeFe] Hydrogenases From O2. *Journal of the American Chemical Society* **2018**, *140* (30), 9346-9350. DOI: 10.1021/jacs.8b04339.
(16) Kisgeropoulos, E. C.; Ratzloff, M. W.; Stroeva-Dahl, E. M.; Hasan, S.; Varghese, F.; Artz, J. H.; Peters, J. W.; Mulder, D. W.; King, P. W. H-cluster Intermediates and Catalytic Properties of Clostridium pasteurianum [FeFe]-Hydrogenase III. *Biochemistry* **2025**. DOI: 10.1021/acs.biochem.5c00066.
(17) Ghosh, S.; Das, C. K.; Uddin, S.; Stripp, S. T.; Engelbrecht, V.; Winkler, M.; Leimkühler, S.; Brocks, C.; Duan, J.; Schäfer, L. V.; et al. Protein Dynamics Affect O2-Stability of Group B [FeFe]-Hydrogenase from Thermosediminibacter oceani. *Journal of the American Chemical Society* **2025**, *147* (18), 15170-15180. DOI: 10.1021/jacs.4c18483.
(18) Greening, C.; Biswas, A.; Carere, C. R.; Jackson, C. J.; Taylor, M. C.; Stott, M. B.; Cook, G. M.; Morales, S. E. Genomic and metagenomic surveys of hydrogenase distribution indicate H2 is a widely utilised energy source for microbial growth and survival. *The ISME Journal* **2016**, *10* (3), 761-777. DOI: 10.1038/ismej.2015.153.
